# Effects of Physical Activity on Brain White Matter Integrity Are Region‐, Dose‐, and Age‐Dependent: Findings From the Population‐Based Rhineland Study

**DOI:** 10.1111/acel.70659

**Published:** 2026-08-07

**Authors:** Eva Saxler, Fabienne A. U. Fox, Alexandra Koch, Xingwang Yang, Weiyi Zeng, Santiago Estrada, Monique M. B. Breteler, N. Ahmad Aziz

**Affiliations:** ^1^ Population Health Sciences German Centre for Neurodegenerative Diseases (DZNE) Bonn Germany; ^2^ Department of Child and Adolescent Psychiatry University of Groningen, University Medical Center Groningen Groningen the Netherlands; ^3^ Accare Child Study Center Groningen the Netherlands; ^4^ The Research School of Behavioural and Cognitive Neurosciences University of Groningen Groningen the Netherlands; ^5^ Artificial Intelligence in Medical Imaging German Center for Neurodegenerative Diseases (DZNE) Bonn Germany; ^6^ Institute for Medical Biometry, Informatics and Epidemiology (IMBIE) University of Bonn, University Hospital Bonn Bonn Germany; ^7^ Centre of Neurology, Clinic for Parkinson, Sleep & Movement Disorders University of Bonn, University Hospital Bonn Bonn Germany

**Keywords:** brain health, diffusion tensor imaging, healthy aging, movement, physical activity, white matter

## Abstract

White matter integrity is a sensitive marker of cerebrovascular health and aging. Physical activity may promote white matter health, but previous studies were limited by self‐reported activity and small sample sizes. We examined the association between accelerometer‐based physical activity and novel indices of white matter integrity while identifying potential mediators. We analyzed cross‐sectional baseline data from 4188 participants (58% women, mean age: 55.4 years) of the Rhineland Study, a prospective cohort study in Bonn, Germany. Physical activity measures (independent variables) were derived from activPAL3 micro‐accelerometers. Fractional anisotropy, mean diffusivity, free water fraction, orientation dispersion, and neurite density (outcomes) were obtained from 3 T MR images for the whole brain and across 27 white matter tracts. We performed multivariable polynomial regression and mediation analyses for cardiometabolic and inflammatory factors. Higher energy expenditure was related to lower orientation dispersion, with the association leveling off at higher amounts (standardized *β*_linear_ = −0.153 [−0.183; −0.122]; *β*_quadratic_ = 0.034 [0.017; 0.051]; both *p* < 0.001). Physical activity was linked to lower neurite density in motor tracts (*β*s between −0.133 and −0.056, all *p* < 0.001). However, with advancing age, physical activity became more strongly associated with higher neurite density and lower mean diffusivity of supratentorial fibers. Body weight, cardiometabolic, and inflammatory factors partly mediated effects of physical activity on white matter microstructure. Physical activity is related to white matter integrity in a nonlinear, age‐ and region‐specific way. Physical activity's effects are greatest in sedentary and older individuals, likely mediated through its impact on body composition, cardiometabolic health, and systemic inflammation.

## Graphical abstract

Physical activity is related to more aligned white matter fibers and microstructural reorganization of the brain. Body weight, cardiometabolic, and inflammatory factors could partly mediate the effects of physical activity on brain white matter microstructure. The effects of physical activity on the brain are age‐ and region‐specific.

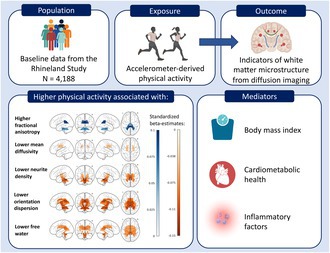

## 1 Introduction

Physical activity (PA) promotes cardiovascular (Kraus et al. 2019) and neuronal (Domingos et al. 2021) health, and it has been associated with a decreased risk of neurodegenerative diseases (López‐Ortiz et al. 2023). In a previous study, we established a link between higher levels of PA and larger gray matter volume (Fox et al. 2022). Building upon this, evaluating brain structural connectivity could enable a better understanding of the effects of PA on brain organization and function (Won et al. 2021).

White matter (WM) integrity, which is crucial for brain structural connectivity, declines with age (Cox et al. 2016) and is affected by neurodegenerative diseases (Rosbergen et al. 2024) as well as by cardiovascular risk (de Groot et al. 2015). Alterations at the microstructural level often precede macrostructural changes in WM (de Groot et al. 2013). PA, on the other hand, has been associated with improved macro‐ and microstructural WM integrity (Sexton et al. 2016; Won et al. 2021). However, previous studies have been hampered by small sample sizes and self‐reported measures of PA, which are subjective and prone to recall bias.

Diffusion‐weighted magnetic resonance imaging (dMRI) allows for measuring the microstructural integrity of WM, which is considered a sensitive marker of cerebrovascular health (de Groot et al. 2015). The structural boundaries of myelinated axons restrict the diffusion of water molecules so that they are more likely to diffuse along, rather than perpendicular to, healthy WM fibers. Diffusion Tensor Imaging (DTI; Basser et al. 1994) utilizes these boundaries to derive measures of WM microstructure such as fractional anisotropy (FA) and mean diffusivity (MD). While FA estimates the proportion of diffusion along one WM fiber tract and reflects greater fiber integrity, MD estimates the overall diffusivity and represents a breakdown in fiber integrity. However, DTI measures may not be sufficiently informative in regions of crossing fibers (Timmers et al. 2016). Neurite orientation and dispersion imaging (NODDI; Zhang et al. 2012) is a biophysical model which describes diffusivity properties of distinct tissue compartments based on dMRI acquisitions of greater diffusion weighting (higher and multiple *b*‐values), and attempts to provide more insight into the biological processes underlying microstructural changes through estimation of the neurite density index (NDI), angular variation of fibers (orientation dispersion index, ODI), free water fraction (isotropic volume fraction, ISOVF), and diffusion from the extra‐neurite space (extracellular volume fraction, ECVF; Timmers et al. 2016).

Here we sought to investigate the effects of accelerometer‐derived PA on WM integrity in a large population‐based cohort study. For a comprehensive assessment of WM health, we leveraged a combination of macrostructural measures as well as measures obtained from both the DTI and the NODDI model. To disentangle the mechanisms through which PA affects WM health, we assessed to which extent cardiometabolic and inflammatory factors mediated the effects of PA on WM microstructure.

## 2 Methods

### 2.1 Study Participants

This study was based on cross‐sectional baseline data from the first 8314 participants (age range: 30–94 years) of the Rhineland Study, an ongoing community‐based prospective cohort study (Ward et al. 2020). Data were collected from March 2016 to November 2021. Invitations to participate were sent to inhabitants of two municipal districts in Bonn, Germany. To enroll in the study, participants were required to have a sufficient command of the German language and be 30 years or older.

Our analytical sample consisted of a subset of 4188 participants with complete actimetry and brain imaging data. Actimetry data of 2154 participants were not available due to the following reasons: refusal to participate (*n* = 141), technical/acquisition failure (*n* = 622), compliance (*n* = 799), ineligibility (*n* = 256), unknown (*n* = 118), and less than five valid days of recording (*n* = 218). Ineligibility criteria and classification of valid days have been reported previously (Fox et al. 2022). Additionally, we excluded 1930 participants who did not have imaging data and 42 participants with missing base covariate data (education level). In models controlling for cardiometabolic risk factors, 54 additional individuals with missing data were excluded (Figure S1).

### 2.2 Standard Protocol Approvals, Registrations, and Patient Consents

The study was approved by the Medical Ethics Committee of the University of Bonn and followed the recommendations of the International Council for Harmonization (ICH) Good Clinical Practice (GCP) standards (ICH‐GCP). Participants provided informed consent in accordance with the principles of the Declaration of Helsinki.

### 2.3 Physical Activity Measurement

PA was measured using the activPAL3 micro‐accelerometer (PAL Technologies, Glasgow, UK), a small (55 × 25 × 5 mm) and lightweight (9 g) triaxial accelerometer with a sampling frequency of 20 Hz. Based on acceleration across the vertical, anteroposterior, and mediolateral axes, the activPAL can be used to estimate energy expenditure and posture (sitting/lying, standing, or stepping) across time. The exact procedure has been described elsewhere (Fox et al. 2022). We primarily focused on average daily energy expenditure in metabolic equivalents per hour (METs) as a proxy for overall PA. To further explore associations of specific subtypes of PA with WM health, we subsequently analyzed daily step count, percent of daily time spent in sedentary (sitting/lying posture; %SED), light‐intensity (standing or step‐taking posture and METs < 3.0, %LIT), or moderate‐to‐vigorous activity (standing or step‐taking posture and METs ≥ 3.0, %MVPA). Weighted daily average values of the variables were calculated across the recording period for each participant to adjust for accelerometer wear time per day, using the following formula:

$$\frac{\sum_{i=1}^{k}\text{daily}\;{\text{hours worn}}_{i}\times \text{daily average of physical}\;{\text{activity measure}}_{i}}{\sum_{i=1}^{k}\text{daily}\;{\text{hours worn}}_{i}}$$

$$k=\text{number of valid days}\;\left(i.e.,5,6,\text{or}\;7\right).$$

### 2.4 Brain MR Imaging

Eligible participants underwent MRI on a 3 Tesla scanner (MAGNETOM Prisma, Siemens Healthineers, Erlangen, Germany) equipped with an 80 mT/m gradient system and a 64‐channel phase‐array head–neck coil. The Rhineland Study MRI protocol has been extensively detailed previously (Koch et al. 2025). In this study, we utilized whole‐brain T1‐weighted, T2‐weighted, T2‐weighted fluid‐attenuated inversion recovery (FLAIR), and diffusion‐weighted images. The T1 protocol used a multi‐echo magnetization‐prepared rapid gradient‐echo (MPRAGE) sequence with two‐dimensional acceleration (acquisition time (TA) = 6.5 min; echo time (TE) = 1.68, 3.29, 4.90, and 6.51 ms; repetition time (TR) = 2560 ms; inversion time (TI) = 1100 ms; flip angle = 7°). The T2 protocol used a three‐dimensional turbo spin‐echo (TSE) sequence with variable flip angles (TA = 4.6 min; TR/TE = 2800/405 ms). Both protocols were acquired at 0.8 mm isotropic resolution. FLAIR images were acquired at 1.0 mm isotropic resolution (TA = 4.5 min; TR/TE = 5000/393 ms; TI = 1800 ms). Diffusion‐weighted MRI scans were acquired at 1.5 mm isotropic resolution using a spin‐echo echo‐planar imaging sequence with threefold slice acceleration and adaptive combination (TA = 12.1 min; TR/TE = 5500/105 ms; multiband factor = 3; maximum *b*‐value = 6800 s/mm^2^; Δ = 51.3 ms; δ = 20.1 ms). Additionally, four pairs of anterior‐to‐posterior and posterior‐to‐anterior phase‐encoded scans were acquired at *b* = 0 s/mm^2^.

We assessed total brain volume (TBV), total white matter volume (WMV), and total volume of white matter hyperintensities (WMHv) as macrostructural indicators of brain‐ and WM health while controlling for estimated total intracranial volume (ETIV). TBV, WMV, and ETIV were quantified using the standardized FreeSurfer 6.0 pipeline (Fischl et al. 2002). For WMHv, we automatically outlined white matter hyperintensities using an in‐house developed deep learning pipeline (Lohner et al. 2022), which integrates information from T1‐weighted, T2‐weighted, and FLAIR images.

We quantified microstructural WM health based on DTI‐ and NODDI‐derived dMRI measures determined across the whole brain and within tract‐specific skeletonized normal‐appearing white matter (NAWM): Preprocessing of the dMRI data in the Rhineland Study has been introduced previously by (Tobisch et al. 2018) and includes correction for head motion (Andersson and Sotiropoulos 2016) as well as susceptibility‐induced and eddy current‐induced geometric distortions (Andersson et al. 2003), followed by compressed sensing reconstruction for accelerated dMRI acquisitions. Subsequently, dMRI metrics were estimated through voxel‐wise model fitting using the Microstructure Diffusion Toolbox (Harms et al. 2017): FA and MD from the DTI model (Basser et al. 1994), and NDI, ODI, and ISOVF from the NODDI model (Zhang et al. 2012). To generate the NAWM mask, we corrected FreeSurfer's whole brain WM mask by removing WMH voxels identified using the aforementioned WMH pipeline. This NAWM mask was further refined through skeletonization and applied to compute the dMRI metrics FA, MD, NDI, ODI, and ISOVF as the average across voxels of the whole brain, as well as separately for 27 white matter tracts from the JHU‐ICBM‐DTI‐81 atlas (Mori et al. 2008).

### 2.5 Covariates

Participants' age and sex were determined based on self‐reports. Using the International Standard Classification of Education 2011, participants' highest educational level was classified as low (lower secondary education or below), middle (upper secondary education to undergraduate university level), and high (postgraduate university study). Current smoking status was binarized into smokers and non‐smokers based on cotinine metabolite levels. Body mass index (BMI) was calculated as weight in kilograms divided by height in meters squared. Waist‐to‐hip ratio (WHR) was calculated as a ratio of waist circumference to hip circumference. Resting blood pressure was measured three times with 10‐min intervals and average systolic blood pressure (SBP) and diastolic blood pressure (DBP) were calculated using the last two measured values. Mean arterial pressure (MAP) was calculated as follows: MAP = (SBP + 2 × DBP)/3. Hypertension was categorized into a binary (Yes/No) variable, where “Yes” was given if participants met at least one of the following criteria: (1) SBP ≥ 140 and/or DBP ≥ 90, (2) regular intake of antihypertensive medication, (3) self‐reported hypertension. Serum levels of cholesterol, high‐density lipoprotein (HDL), low‐density lipoprotein (LDL), C‐reactive protein (CRP), insulin levels, and triglycerides were measured using routine methods at the Clinical Chemistry Laboratory of University Hospital Bonn. Participants' medical history of neurological disorders (including dementia, Parkinson's disease, multiple sclerosis, stroke, and transient ischemic attack) was obtained based on self‐reports and categorized as a binary variable (Yes/No).

### 2.6 Statistical Analysis

Multiple polynomial regression models were used to assess the association between PA (independent variable) and brain structure (outcome). We defined three consecutive models: the base model included age, sex and education as covariates (Model 1). In Model 2, we additionally included quadratic terms for age and PA to assess potential non‐linear associations, while in Model 3, we also included an interaction term between PA and age. Finally, as a sensitivity analysis, we ran another model which additionally controlled for three common cardiometabolic risk factors: smoking, hypertension, and BMI (Model 4). Higher‐order terms (i.e., quadratic terms for age and PA, and PA × age interaction terms) were only retained in the next model if they reached statistical significance (*p* ≤ 0.05).

All continuous variables were z‐standardized for better comparability of effect sizes. Distributions of all dependent variables were checked visually. All variables except for MD values of two tracts (pontine crossing tract and medial lemniscus) followed a normal distribution. Due to skewed distributions, regression analyses for these tracts were re‐run with log‐transformed variables and compared. The final results did not change substantially, so the results are reported based on the untransformed variables for ease of interpretation. All residual and quantile‐quantile plots were examined visually. Cook's distance was computed for all models to screen for influential points (all < 1). Diffusion data of the 27 tracts were averaged over hemispheres. If data of one hemisphere was missing, values of the available hemisphere were used. As the diffusion data across different metrics and tracts cannot be seen as independent from one another, we corrected for multiple comparisons by first estimating the effective number of independent tests (Meff), which accounts for the correlation between hypotheses as described previously (Li and Ji 2005). For the global diffusion metrics, Meff was derived from the correlation matrix of all five diffusion metrics and equaled four. For the tract‐specific analyses, Meff was derived from the correlation matrix of all five diffusion metrics for each of the 27 tracts (135 variables) and equaled 68. Through Li and Ji's correction, this resulted in a corrected threshold for statistical significance of *p*_c_ = 0.013 for global diffusion metrics and *p*_c_ = 0.0008 for tract‐specific analyses (Li and Ji 2005). All statistical analyses were conducted using R (base version 4.3.2).

### 2.7 Sensitivity Analyses and Other Measures of PA as Predictors

In addition to adjusting for cardiometabolic risk factors in a fourth model (see above), all models were re‐run based on a sample from which outliers and participants with a history of neurological disorders were excluded (*n* = 3912, Figure S2). In exploratory analyses, we also assessed associations of four other PA measures (step count, %SED, %LIT, %MVPA) with WM health in the main sample using the same approach as detailed above.

We additionally repeated our analyses in regional WM microstructure for total WM (instead of NAWM). Finally, as a quality control, we included ECVF as an outcome in the models. Based on the model definition of NODDI, this parameter should show opposite effects from NDI. As it is redundant, we have therefore not included it in the main analyses.

### 2.8 Mediation Analyses

**FIGURE 1:**
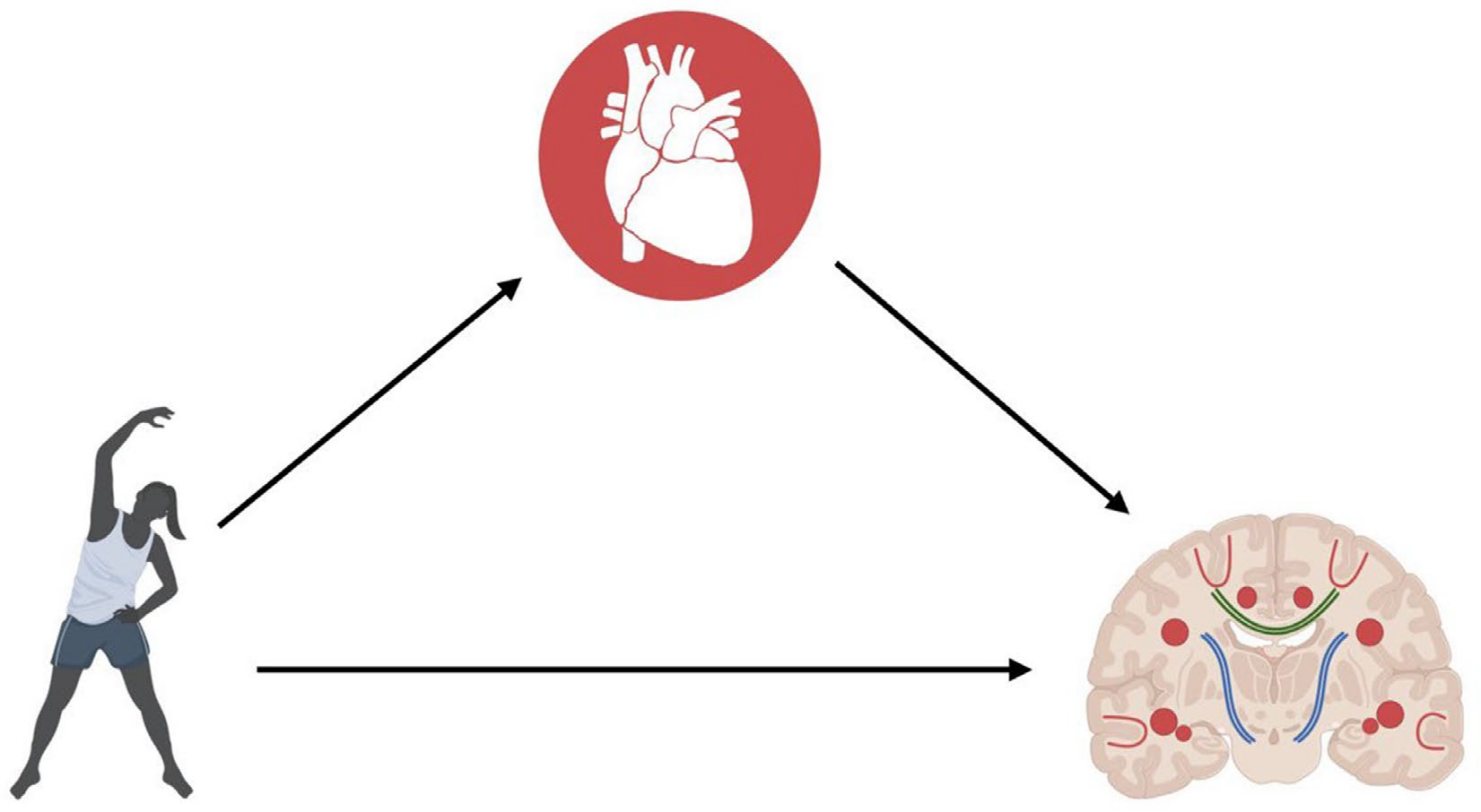
Mediation analysis. *Predictor*: Physical activity was operationalized as energy expenditure (average weighted daily metabolic equivalents). *Mediator* was either the factor analysis‐based cardiometabolic risk score or one individual variable (either body mass index, blood pressure, or C‐reactive protein). *Outcome* was one indicator of white matter microstructure, averaged over the whole brain skeletonized normal appearing white matter (fractional anisotropy, neurite density index, or orientation dispersion index). Images created with biorender.

We examined whether the association between PA and WM microstructure was mediated through cardiometabolic risk or systemic inflammation (Figure 1). For PA we focused again on energy expenditure (i.e., METs), and as outcomes we used the diffusion parameters that were averaged over the whole brain skeletonized NAWM and reached statistical significance in regression analyses (global mean FA, NDI, ODI, and ISOVF). For cardiometabolic risk, we used single variables as mediators (BMI and MAP), as well as sample‐based (factor analysis for mixed data (FAMD)‐derived) cardiometabolic disease risk components. FAMD was performed using the FactoMineR package in R and based on 10 variables: DBP, SBP, hypertension, WHR, triglycerides, cholesterol, HDL, LDL, insulin, and smoking status. CRP was used as a proxy for systemic inflammation and log‐transformed due to skewed distribution. Mediation analyses were performed through structural equation modeling using the lavaan package in R. All mediation models were adjusted for age, age^2^, sex, and education. Given the non‐linear association of physical activity with cardiometabolic risk and WM microstructure, we present estimates at the average, 1 standard deviation (SD) below, and 1 SD above mean PA dose. Quadratic PA terms were only added to the final models if they were statistically significant (*p* ≤ 0.05).

### 2.9 Role of the Funding Source

The funding source had no involvement in study design, data collection, data analysis and interpretation, writing, or in the decision to submit the paper for publication.

## 3 Results

The mean (SD) age in our analytical sample was 55.4 (13.4) years, ranging from 30 to 94. The sample included 58% females and participants with a relatively high education level (Table 1). Compared to men, women had a slightly higher energy expenditure (METs), which was likely driven by higher amounts of light intensity PA and lower amounts of time spent sedentary. Mean derived diffusion measures (i.e., FA, ODI, NDI, and ISOVF) were higher in men compared to women. Physically active participants were younger and more often female than physically inactive participants (Table S1). Participants who were included in the analytical sample were on average slightly younger, more often female and had a more favorable health profile (Table S2).

**TABLE 1:** Sample characteristics.

	*N*	Overall, *N* = 4188	Male, *N* = 1777	Female, *N* = 2411	*p*
Age (years)	4188	55.37 (13.42)	55.31 (13.69)	55.41 (13.23)	0.8
Age groups	4188				0.14
30–39		665 (16%)	302 (17%)	363 (15%)	
40–49		690 (16%)	278 (16%)	412 (17%)	
50–59		1199 (29%)	497 (28%)	702 (29%)	
60–69		981 (23%)	402 (23%)	579 (24%)	
70–79		502 (12%)	232 (13%)	270 (11%)	
80–89		147 (3.5%)	63 (3.5%)	84 (3.5%)	
90+		4 (< 0.1%)	3 (0.2%)	1 (< 0.1%)	
Education level (ISCED11)	4188				< 0.001
Low		68 (1.6%)	18 (1.0%)	50 (2.1%)	
Middle		1843 (44%)	624 (35%)	1219 (51%)	
High		2277 (54%)	1135 (64%)	1142 (47%)	
Body mass index (kg/m^2^)	4177	25.596 (4.137)	26.158 (3.441)	25.181 (4.541)	< 0.001
Missing		11	0	11	
Current smoker	4183				0.11
No		3663 (88%)	1537 (87%)	2126 (88%)	
Yes		520 (12%)	238 (13%)	282 (12%)	
Missing		5	2	3	
Hypertension (binary)	4147				< 0.001
No		2707 (65%)	1055 (60%)	1652 (69%)	
Yes		1440 (35%)	708 (40%)	732 (31%)	
Missing		41	14	27	
Systolic blood pressure (mmHg)	4165	125.73 (15.52)	129.53 (13.33)	122.92 (16.40)	< 0.001
Missing		23	3	20	
Diastolic blood pressure (mmHg)	4165	74.65 (9.12)	76.52 (9.03)	73.26 (8.94)	< 0.001
Missing		23	3	20	
Neurological disorders (binary)	4188				0.084
No		4078 (97%)	1721 (97%)	2357 (98%)	
Yes		110 (2.6%)	56 (3.2%)	54 (2.2%)	
Actimetry sensor: sum of hours worn	4188	166.87 (4.54)	166.73 (4.98)	166.98 (4.19)	0.078
Average weighted daily MET hours	4188	34.02 (1.34)	33.92 (1.39)	34.10 (1.30)	< 0.001
Average weighted daily step count	4188	8844.66 (3217.31)	8789.99 (3354.78)	8884.96 (3112.19)	0.4
Average weighted daily % light intensity PA	4188	0.21 (0.06)	0.19 (0.06)	0.23 (0.06)	< 0.001
Average weighted daily % MVPA	4188	0.05 (0.02)	0.05 (0.02)	0.05 (0.02)	0.6
Average weighted daily % sedentary PA	4188	0.74 (0.07)	0.76 (0.06)	0.73 (0.07)	< 0.001
Total brain volume (cm^3^)	4165	1,104,480.49 (116,055.71)	1,176,845.60 (106,559.36)	1,051,209.64 (91,296.26)	< 0.001
Missing		23	11	12	
Total white matter volume (cm^3^)	4174	455,311.83 (57,831.08)	489,614.49 (53,936.05)	430,080.48 (46,510.65)	< 0.001
Missing		14	8	6	
Estimated total intracranial volume (cm^3^)	4175	1,549,909.76 (150,816.04)	1,653,625.66 (131,847.41)	1,473,653.13 (113,871.58)	< 0.001
Missing		13	8	5	
Total volume of supratentorial WMH (cm^3^)	4188	1.73 (4.18)	1.68 (4.04)	1.78 (4.28)	0.4
Global mean FA in skeletonized NAWM	4188	0.617 (0.019)	0.616 (0.019)	0.619 (0.018)	< 0.001
Global mean MD in skeletonized NAWM × e10	4188	7.414 (0.284)	7.422 (0.289)	7.408 (0.279)	0.10
Global mean ODI in skeletonized NAWM	4188	0.217 (0.009)	0.220 (0.009)	0.215 (0.009)	< 0.001
Global mean NDI in skeletonized NAWM	4188	0.669 (0.034)	0.671 (0.034)	0.667 (0.033)	< 0.001
Global mean ISOVF in skeletonized NAWM	4188	0.044 (0.009)	0.045 (0.009)	0.043 (0.009)	< 0.001

*Note:* Categorical data: *n* (%); continuous data: mean (SD); to assess group differences between males and females, a *χ* ^2^ test was used for categorical variables and a 2‐sample *t*‐test for continuous variables.

Abbreviations: FA = fractional anisotropy, ISCED = International Standard Classification of Education, ISOVF = isotropic volume fraction, MD = mean diffusivity, MET = metabolic equvalents, MVPA = moderate‐to‐vigorous PA, NAWM = normal‐appearing white matter, NDI = neurite density index, ODI = orientation dispersion index, PA = physical activity, WMH = white matter hyperintensities.

### 3.1 Global Macrostructure

We report results for the fully adjusted model (Model 3). Increases across all PA measures were nonlinearly associated with higher TBV, where the effect leveled off at higher amounts of PA (Figure S3). More time spent in moderate‐to‐vigorous PA was most robustly associated with lower WMHv, with the association also leveling off at higher activity levels (*β*_linear_ = −0.043 [−0.072; −0.014], *p* = 0.004; *β*_quadratic_ = 0.015 [0; 0.03], *p* = 0.044). Total WMV showed the weakest associations with PA, but small interaction effects indicated that with higher age, more time spent in light PA was more strongly associated with higher WMV (*β*_%LIT×age_ = 0.015 [0.002; 0.028], *p* = 0.029); and more time spent sedentary was more strongly associated with lower WMV (*β*_%SED×age_ = −0.015 [−0.028; −0.002], *p* = 0.025).

### 3.2 Global Microstructure

**FIGURE 2:**
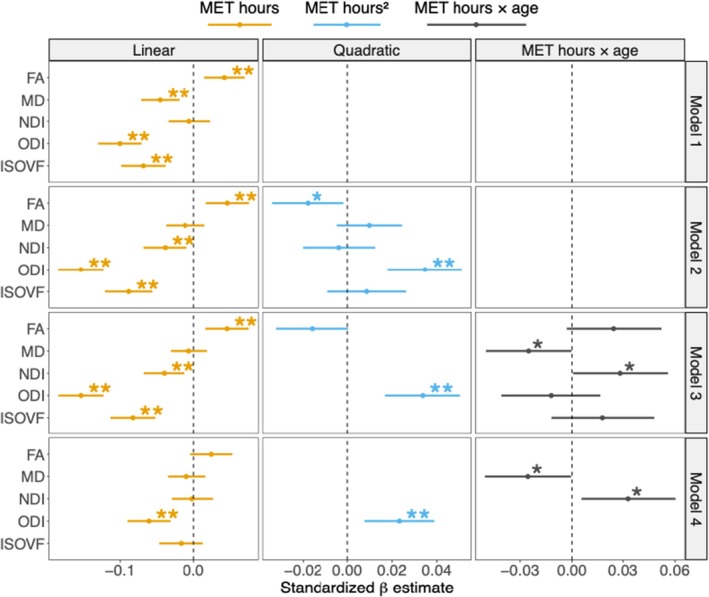
Effects of metabolic equivalents per hour (MET hours) on global white matter microstructure. *p < 0.05; **p < *p*_c_. FA = fractional anisotropy, ISOVF = isotropic volume fraction, MD = mean diffusivity, MET = metabolic equivalents, NDI = neurite density index, ODI = orientation dispersion index, *p*_c_ = Ji & Li‐corrected level of statistical significance (= 0.013).

Higher energy expenditure was associated with higher global FA (*β*_linear_ = 0.045 [0.016; 0.075], *p* = 0.002), lower global mean neurite density (*β*_linear_ = −0.040 [−0.068; −0.012], *p* = 0.005), lower overall fanning of fibers—particularly at the lower end of the PA spectrum (mean global ODI regressed on METs: *β*_linear_ = −0.153 [−0.183; −0.122], *p* < 0.0001; *β*_quadratic_ = 0.034 [0.017; 0.051], *p* < 0.0001) –, and lower ISOVF (*β*_linear_ = −0.083 [−0.113; −0.053], *p* < 0.0001; Figure 2).

Other PA measures exhibited similar associations with global microstructure, with light PA showing the weakest associations and sedentary activity showing the opposite directions of effects (e.g., higher ODI with more time spent sedentary; Figure S4). Additionally, increased time spent in moderate‐to‐vigorous PA was associated with higher FA especially in people of older age (*β*_%MVPA×age_ = 0.036 [0.008; 0.063], *p* = 0.012).

### 3.3 Regional Microstructure

**FIGURE 3:**
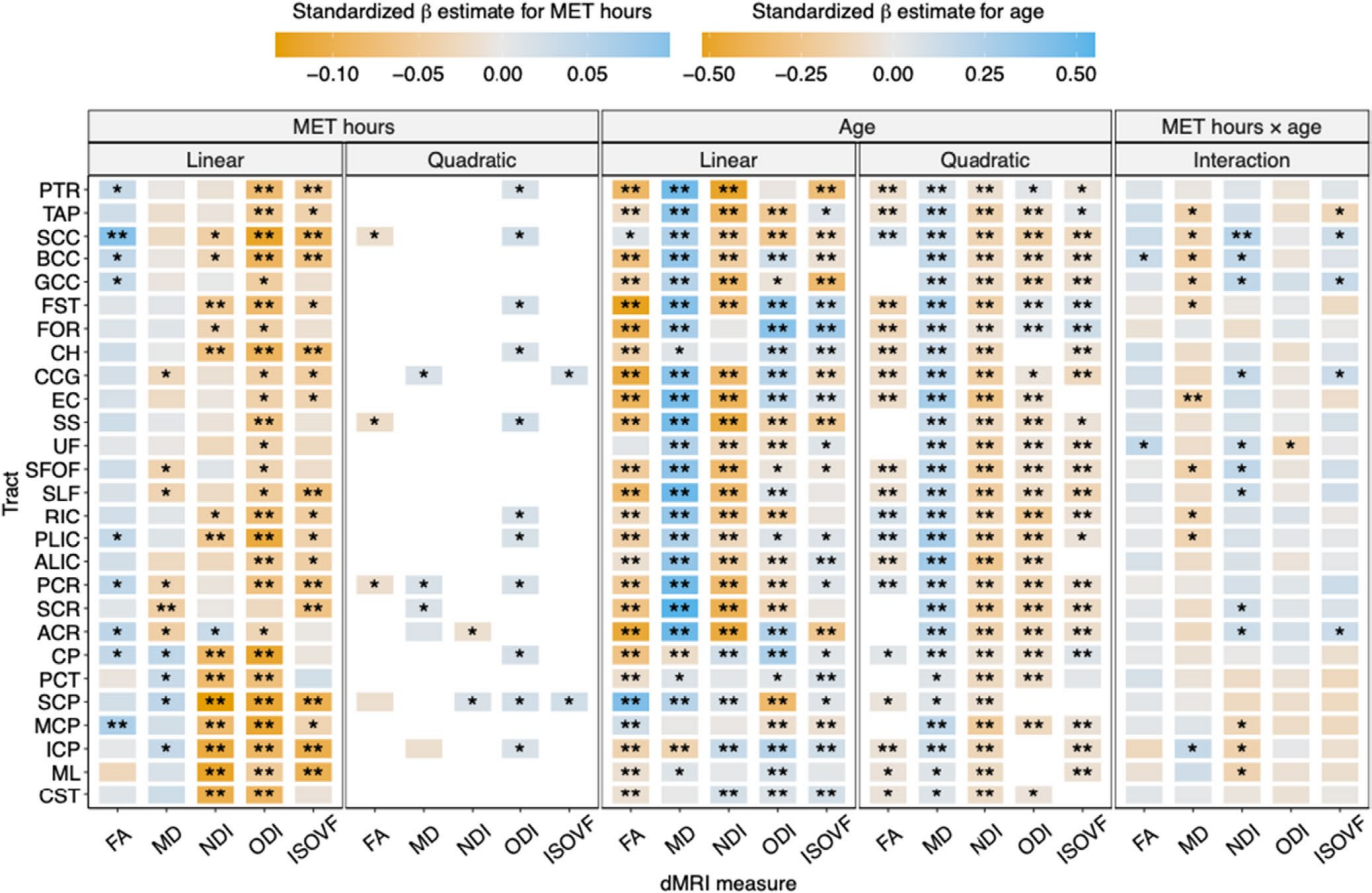
Effects of metabolic equivalents per hour (MET hours) and age on regional white matter microstructure. The beta estimates are derived from the final model, after controlling for age, sex, education, PA × age and, where applicable, for age^2^ and PA^2^. White spaces indicate the instances where the higher order term failed to reach significance and was omitted from the final model. Legend for MET hours refers to linear, quadratic, and interaction effects of MET hours. *p < 0.05; **p < *p*_c_. ACR = anterior corona radiata, ALIC = anterior limb of internal capsule, BCC = body of corpus callosum, CCG = cingulum/cingulate gyrus, CH = cingulum hippocampus, CP = cerebral peduncle, CST = corticospinal tract, dMRI = diffusion‐weighted MRI, EC = external capsule, FOR = fornix, FST = fornix/stria terminalis, GCC = genu of corpus callosum, ICP = inferior cerebellar peduncle, MCP = middle cerebellar peduncle, MET = metabolic equivalents, ML = medial lemniscus, PCR = posterior corona radiata, PCT = pontine crossing tract, PLIC = posterior limb of internal capsule, PTR = posterior thalamic radiation, RIC = rostral limb of internal capsule, SCC = splenium of corpus callosum, SCP = superior cerebellar peduncle, SCR = superior corona radiata, SFOF = superior fronto‐occipital fasciculus, SLF = superior longitudinal fasciculus, SS = sagittal striatum, TAP = tapetum, UF = uncinate fasciculus, *p*_c_ = Li & Ji‐corrected level of statistical significance (= 0.0008).

**FIGURE 4:**
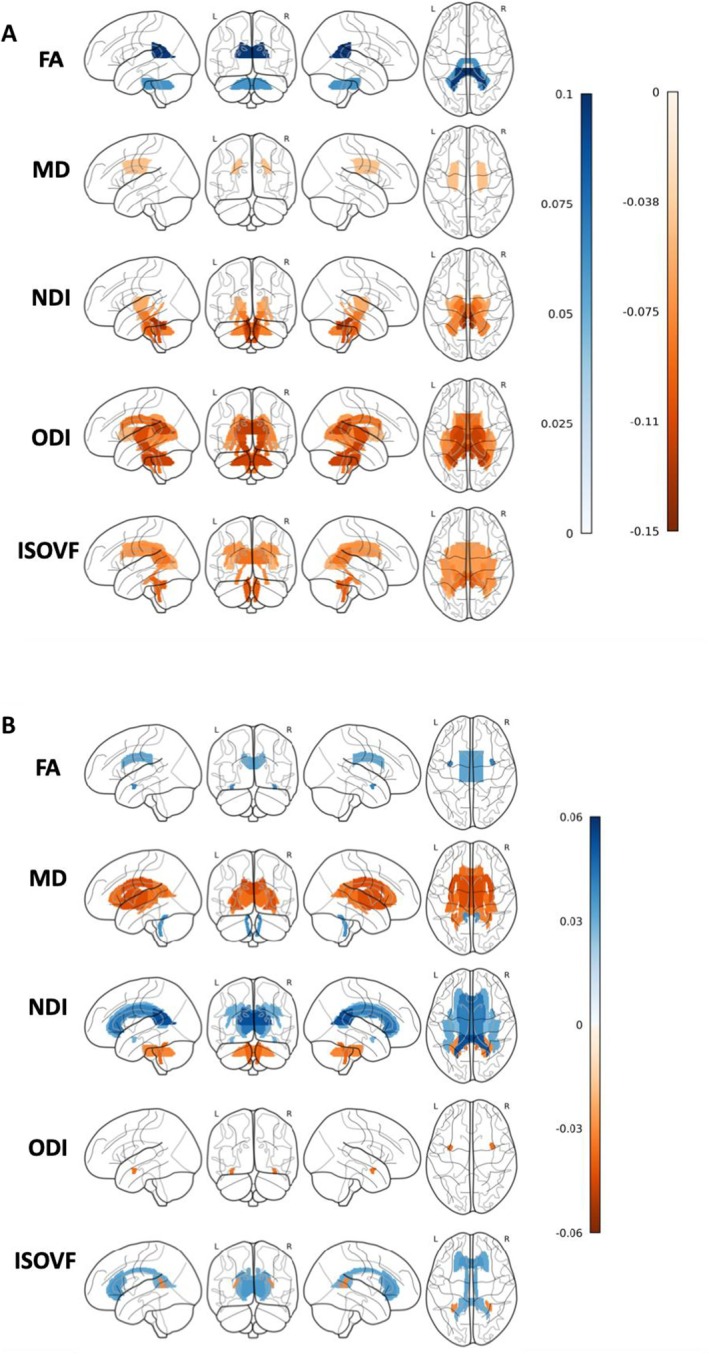
Effects of increased metabolic equivalents per hour (MET hours) on white matter tracts. (A) Main effects of MET hours on dMRI parameters. All *p*‐values survived correction for multiple comparisons. (B) Interaction effects of MET hours on dMRI parameters. With increasing age, the effect of MET hours became stronger. All *p*‐values are < 0.05. DMRI = diffusion MRI, FA = fractional anisotropy, ISOVF = isotropic volume fraction, MD = mean diffusivity, MET = metabolic equivalents, NDI = neurite density index, ODI = orientation dispersion index.

Regarding the tract‐specific analyses, most widespread associations were observed between energy expenditure and ODI, where higher METs were related to lower fanning of fibers (Figures 3 and 4). Energy expenditure was also related to lower neurite density; however, this was mainly confined to motor tracts and fibers close to the brainstem (corticospinal tract, medial lemniscus, cerebellar peduncles, pontine crossing tract, cerebral peduncle, posterior limb of internal capsule), as well as to the fornix/stria terminalis and cingulum/hippocampus. Some of these regions additionally showed lower free water content with increasing energy expenditure (medial lemniscus, inferior and superior cerebellar peduncles, cingulum/hippocampus). Parameters from the DTI model exhibited weaker associations than parameters from the NODDI model (Figures 3 and 4). As depicted in Figure 3, higher FA in the splenium of the corpus callosum was associated with higher energy expenditure (*β*_linear_ = 0.097 [0.065; 0.129], *p* < 0.0001). Also, higher METs were related to higher FA of the middle cerebellar peduncle (*β*_linear_ = 0.061 [0.031; 0.092], *p* < 0.0001) and lower MD of the superior corona radiata (*β*_linear_ = −0.045 [−0.070; −0.019], *p* < 0.0001). Results for Model 1, 2, and 4 are displayed in Figures S5, S6, and S8, respectively.

Other PA measures yielded similar results (Figure S7).

**FIGURE 5:**
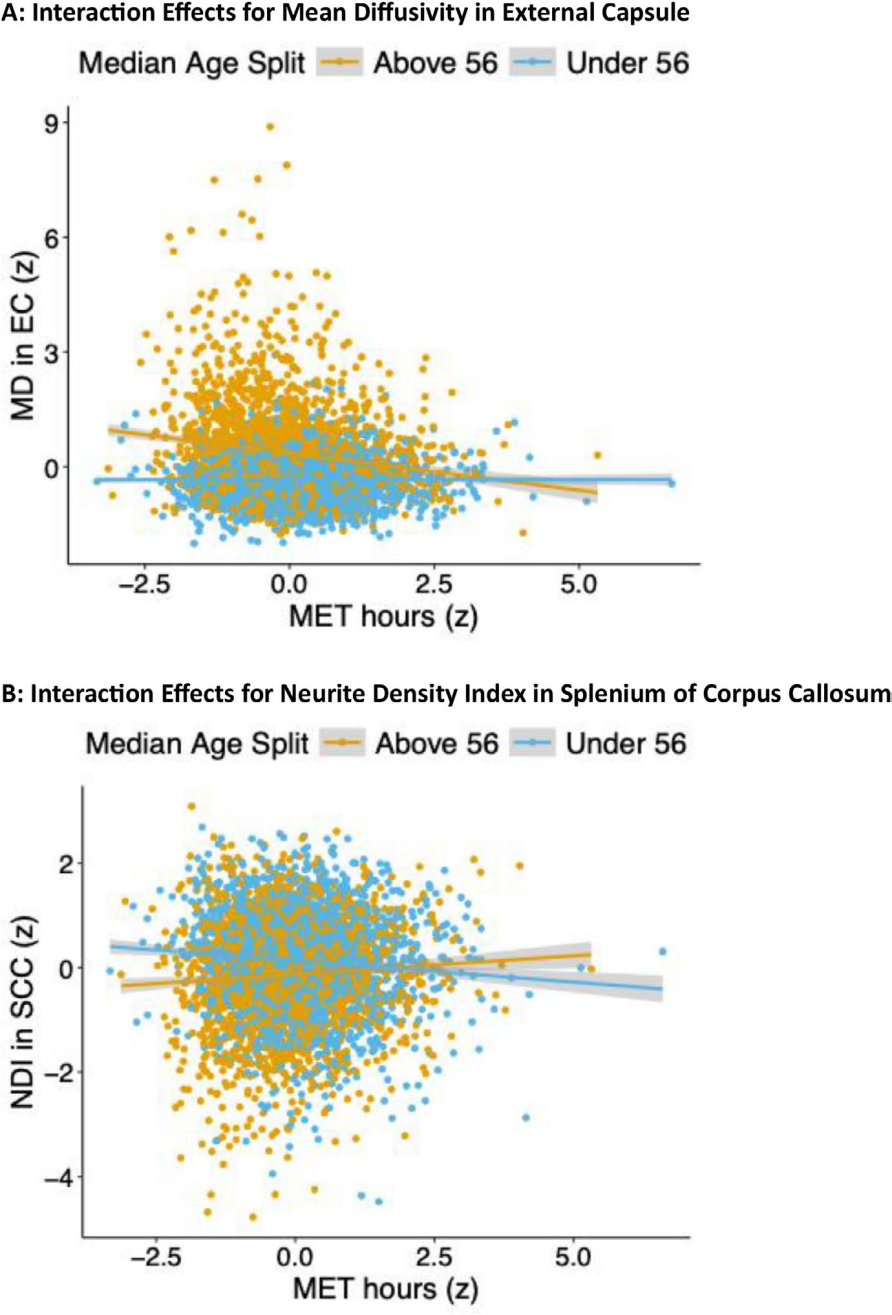
Interaction effects between MET hours and age. Interaction effects between MET hours and age, which survived correction for multiple comparisons. For individuals of higher age, increased MET hours were associated with lower MD in the external capsule (A) and with higher NDI (B) in the splenium of corpus callosum. Median split by age was only performed for illustrative purposes. Analyses were based on continuous data. EC = external capsule, MD = mean diffusivity, MET = metabolic equivalents, NDI = neurite density index, SCC = splenium of corpus callosum.

Furthermore, nominally significant interaction effects indicated that with increasing age, PA became associated with higher NDI and lower MD of several association‐ and commissural fibers (Figures 3 and 4; Figures S7, S9, and S10). Interaction effects of energy expenditure and age on MD of the external capsule, as well as on NDI of the splenium of the corpus callosum survived correcting for multiple comparisons (MD: *β*_MET×age_ = −0.044 [−0.069; −0.019], *p* = 0.0005; NDI: *β*_MET×age_ = 0.053 [0.023; 0.082], *p* = 0.0005; Figure 5A,B).

For older individuals, the direction of associations between PA and NDI in association and commissural fibers was opposite to those of the age‐NDI associations, whereas for NDI in motor tracts, we could not observe opposite directions of associations for age and PA in older age (Figures 3, 4, 5, Figures S9 and S11).

### 3.4 Sensitivity Analyses

Additional controlling for cardiometabolic risk factors (Model 4) attenuated the associations of PA with microstructural parameters (Figures S4 and S8) more than on macrostructural parameters (Figure S3). Global mean ODI remained associated with PA after controlling for cardiometabolic risk (e.g., METs: *β*_linear_ = −0.061 [−0.090; −0.032], *p* < 0.0001; *β*_quadratic_ = 0.023 [0.008; 0.039], *p* = 0.003), whereas associations of PA with overall FA, NDI, and ISOVF became statistically non‐significant (Figure 2; Figure S4). Looking into regional microstructure, however, beta estimates of PA were mitigated most for ODI, as the widespread associations were now confined to a smaller number of tracts (Figure S8). The interaction effects of PA and age on MD and NDI of individual tracts were affected least by controlling for cardiometabolic risk factors (Figure S8).

After removing outliers and participants with neurological diseases, no associations between energy expenditure and macrostructural measures remained in the fully adjusted Model (Figure S12). Associations of energy expenditure with global mean microstructure did not change substantially (Figure S13). Likewise, most of the tract‐specific results did not change substantially (Figures S14–S17); however, the interaction effects of energy expenditure and age on NDI in the splenium of the corpus callosum and on MD of the external capsule no longer reached corrected statistical significance (Figure S16). Attenuation of macrostructural findings was mostly driven by excluding outliers, whereas attenuation of interaction effects on WM microstructure was mainly driven by excluding participants with neurological diseases (data not shown).

Results of our quality control were as expected: The ECVF‐associations with PA yielded the same results as the NDI‐associations, with the beta estimates pointing in the opposite direction (data not shown).

Analyses in total WM were not meaningfully different from analyses in NAWM (Figure S18). Associations of energy expenditure with MD of the superior and posterior corona radiata were slightly greater, whereas the association with FA of the superior fronto‐occipital fasciculus was slightly less pronounced in NAWM compared to WM (Table S3).

To compare associations between energy expenditure and dispersion across tracts with higher and lower ODI, we sorted fibers by mean ODI and looked at the regression coefficients in all tracts (Tables S4 and S5). Three of our top hits were also three of the more aligned tracts (splenium of the corpus callosum, superior cerebellar peduncle, and posterior limb of the internal capsule), which suggests that PA could especially support a coherent fiber structure in these regions. Beyond this; however, post hoc analyses to assess associations between ODI and CRP in all tracts yielded more widespread effects: ODI was related to higher CRP levels in most tracts (Figure S19).

### 3.5 Mediation Analyses

For the sample‐based score, we specifically assessed the mediation effect of the first and second FAMD cardiometabolic component, which were predominantly influenced by (1) blood pressure and adiposity measures, and (2) blood lipid levels, respectively (Figure S20A,B). Diffusion parameters from the NODDI model showed greater mediation effects than those from the DTI model (Table S6). ODI revealed the strongest mediation effects, which became progressively stronger at higher energy expenditure: for example, keeping the covariates at a constant, component 1 from FAMD mediated 15%, 22%, and 41% of the effects of PA on global ODI at low, average, and high energy expenditure levels, respectively.

BMI was the strongest mediator of the effects of PA: At low quantities of energy expenditure (−1 SD), 87% of the effects of energy expenditure on global NDI were mediated by BMI, with an indirect effect of −0.05. The mediated proportions became smaller with more energy expenditure, but remained substantial (84% at average energy expenditure, and 80% at 1 SD above the mean energy expenditure). The correlation between global mean NDI and BMI was highly significant (Pearson's *r* = 0.16; *p* < 0.0001; data not shown). However, proportions mediated by BMI were similar for ISOVF (76% at low energy expenditure) and the correlation between BMI and ISOVF was also positive (*r* = 0.31, *p* < 0.0001).

Additional post hoc analyses with CRP as a measure of systemic inflammation revealed that PA's effects on both ODI and NDI were also partially mediated by CRP. While mediation effects became progressively stronger at higher energy expenditure for ODI (mediated proportion reaching 15%, 16%, and 19% at −1 SD, average, and +1 SD, respectively), the mediation effects became progressively weaker at higher energy expenditure for NDI (mediated proportion reaching 35%, 28%, and 20% at −1 SD, average and +1 SD, respectively).

## 4 Discussion

Here we provide a detailed analysis of the associations between accelerometer‐derived PA measures and WM microstructure. We found that: (1) increased PA is consistently associated with lower fanning (ODI) across most WM regions of the brain, which is partly attributable to the effects of PA on the cardiovascular system; (2) the associations are tract type specific, with the strongest relationships between PA and WM microstructure found in motor tracts, cingulum/hippocampus, and the splenium of corpus callosum; and (3) the associations between PA and microstructural integrity become stronger with age.

Global mean ODI decreased with higher PA, with the association leveling off at higher quantities. This pattern was also reflected in widespread, age‐independent decreases in tract‐specific ODI. These findings fit well with (Chen et al. 2025) and are in line with those obtained in a sample of 816 healthy adults (Callow et al. 2022), in whom cardiovascular fitness was related to lower ODI of the motor tracts. Less fanning of fibers (i.e., lower ODI) has been suggested to reflect a greater coherence and integrity of WM (Callow et al. 2022). Additionally, previous studies suggest that higher ODI might correlate positively with microglial density, which could in turn reflect neuroinflammatory processes (Beydoun et al. 2024; Yi et al. 2019). Indeed, an increased number of past viral or bacterial infections has been associated with higher ODI in several WM tracts (Beydoun et al. 2024), which was more pronounced in people with poor cardiovascular health. Additionally, recent findings suggest higher ODI to be associated with increased inflammation markers in blood plasma (Mishra et al. 2026). Cardiovascular fitness and PA might counteract inflammatory processes in WM, suggesting that a negative association between PA and ODI may reflect anti‐inflammatory effects of moderate PA (Chen and Nakagawa 2023; El Assar et al. 2022; Gleeson et al. 2011). In line with this, ODI in most tracts showed positive correlations with blood levels of CRP, a marker of systemic inflammation. Additionally, our mediation analyses revealed that the negative association of PA with global ODI may partly be attributed to reduced levels of CRP with higher PA.

Furthermore, controlling for cardiometabolic risk factors (hypertension, smoking and BMI) confined associations to a smaller number of tracts. This fits well with our mediation analyses, where up to 41% of the effects of PA on ODI were mediated by cardiometabolic risk. A large‐scale study revealed that MAP was positively associated with ODI in tracts supplied by the posterior circulation (e.g., pontine crossing tract, corticospinal tract, cerebral peduncle, medial lemniscus and inferior cerebellar peduncle; Wartolowska and Webb 2022). These tracts also overlap with tracts for which associations no longer reached significance after adjusting for cardiometabolic risk factors in our study, which suggests that control of hypertension may be of particular importance for white matter health.

There are several mechanisms through which cardio‐ and cerebrovascular health could affect WM integrity. Some main pathways include hypoxia, ischemia, and myelin injury as a result of hypertension (Petrea et al. 2024), endothelial dysfunction and increased blood‐brain barrier permeability as a result of hyperglycemia (Sun et al. 2020), as well as the suppression of genes needed for myelin synthesis and maintenance by cigarette smoke (Yu et al. 2016). PA, on the other hand, may offer protective effects, for example, by promoting the release of vascular endothelial growth factor, which drives blood vessel formation (Tari et al. 2025). However, intrinsic limitations of dMRI model parameters such as ODI should be considered when interpreting the results. ODI, by definition, varies between approximately unidirectional and crossing fiber regions within the brain white matter. We observed widespread decreases in ODI with more PA, which were not restricted to unidirectional fibers, as well as relatively widespread associations between greater ODI and higher CRP levels. While these results suggest that *relative* decreases in orientation dispersion of even rather dispersed structures may reflect certain favorable processes in WM microstructure, more detailed studies are required to disentangle the contributions of the underlying microstructural variation and any neurological process to the changes in ODI.

In addition to the above interpretations, reverse‐causation may also partly account for our findings in this cross‐sectional study, that is, healthier individuals are better able to be physically active and are likely to enjoy exercise more than diseased individuals.

Surprisingly, higher PA was associated with lower NDI, especially in motor tracts (e.g., corticospinal tract, medial lemniscus, superior, middle and inferior cerebellar peduncle, pontine crossing tract, cerebral peduncle, posterior limb of internal capsule), largely independent of age. One interpretation could be that lower NDI reflects decreased microstructural integrity. In support of this, a recent study found that marathon running reversibly decreases myelin content in motor fibers, which overlapped with our identified tracts (corticospinal tract, cerebellar peduncles, pontine crossing tract; Ramos‐Cabrer et al. 2025). The authors hypothesize that myelin lipids may act as glial energy reserves in extreme metabolic conditions. It is, however, unlikely that extreme cases such as marathon runners influenced our results meaningfully. Looking at free water, which also decreased with more energy expenditure, it seems more likely that reduced NDI reflects a restructuring of fibers with more activity. For example, lower NDI in motor tracts could indicate an increased capillary density with higher fitness levels (Callow et al. 2022). Decreased NDI by definition of the NODDI model stems from an increased contribution of the extra‐cellular compartment which in case of its association with more PA could be driven by vascular remodeling. In support of this, exercise has been shown to induce angiogenesis in the brain (Chen and Nakagawa 2023; Morland et al. 2017; Swain et al. 2003) and improve cerebral blood flow (Liu et al. 2023; Swain et al. 2003; Tomoto et al. 2023). Our quality check with ECVF confirmed opposite associations for NDI and ECVF. Aside from neurite density itself, NDI could therefore also be influenced by changes in components of ECVF, for example the extracellular matrix (Le Grand et al. 2024).

While CRP and ODI were negatively associated, CRP and overall ECVF were not linked. This supports our speculation that ODI could be related to inflammatory processes, whereas increases in ECVF (and corresponding decreases in NDI) could rather be tied to remodeling of the extra‐neurite space, that is, through angiogenesis. However, these interpretations remain speculative and warrant further investigation.

BMI mediated the majority of the effects of PA on global mean NDI and ISOVF. We found a highly significant positive correlation between NDI or ISOVF and BMI. Hence, PA could affect neuronal restructuring mostly indirectly via reduction of body weight and body fat percentage. An underlying mechanism could be a higher availability of leptin with a higher body fat percentage: Leptin is primarily secreted by adipose tissue and recent studies found that higher leptin availability is related to better preservation of white matter microstructure (Charisis et al. 2024). Moreover, leptin may have neuroprotective effects on dopaminergic neurons in Parkinson's disease (Sharma et al. 2025). On the other hand, a higher cardiovascular risk associated with adiposity could promote inflammatory processes and decreased microstructural integrity reflected in increased free water content. Controlling for cardiometabolic risk factors including BMI in Model 4 attenuated the associations, but the magnitude of the associations in the corticospinal tract, cerebellar peduncles, and medial lemniscus remained largely unchanged, suggesting that some associations between PA and white matter integrity are independent of BMI.

We did not observe associations between PA and NDI or MD of most association‐ and commissural fibers, but several nominally significant interaction effects indicated that PA may relate to WM microstructure of these tracts in an age‐dependent manner. With older age, there was a trend for higher PA becoming associated with lower MD and higher NDI in selected association‐ and commissural fibers. Furthermore, associations of WM integrity with age and PA were pointed in opposite directions for older individuals in these supratentorial tracts: While increasing age was associated with lower NDI and higher MD, respectively, the associations of PA with these measures were in the opposite direction for older ages. Our results are in line with previous reports of more pronounced effects of PA in older individuals (Mace et al. 2021; Won et al. 2021). While this could be due to a ceiling effect in younger people with healthy WM, the nominal PA‐age‐interaction effects in association fibers could also be attributed to the last‐in‐first‐out‐hypothesis (Naftali Raz 2001; N. Raz 2000): Late‐myelinating fibers such as association fibers and anterior commissural fibers are most prone to age‐related degeneration (Cox et al. 2016; Slater et al. 2019; Wassenaar et al. 2019), but at the same time may also be most responsive to prevention through lifestyle interventions (Trofimova et al. 2023; Wassenaar et al. 2019).

### 4.1 Limitations

Our study was based on cross‐sectional baseline data, which did not allow the assessment of the directionality of the associations. Even though recordings were made during regular activity weeks to ensure representativeness, participants may have consciously or unconsciously adapted their PA levels. Finally, our participants were highly educated and comparably physically active, so our findings may have partly been influenced by selection bias. However, focusing on a relatively physically active sample is more likely to have resulted in an underestimation of the effects of physical activity on white matter health as the strongest associations were seen in the lower ranges.

## 5 Conclusion

In conclusion, we found that PA is associated with WM integrity in a nonlinear, age‐ and region‐specific way, indicating that sedentary and older individuals may benefit most from additional PA. While the integrity of motor tracts was consistently associated with PA in all individuals, the association of commissural fibers was most strongly linked to PA in older individuals. Increased PA was consistently associated with lower fanning (ODI) across many WM tracts, possibly attributable to better cardiometabolic health and reduced inflammation. Conversely, higher amounts of PA were associated with lower NDI, especially in motor tracts, which was largely attributable to BMI. However, lower NDI might also suggest a lower myelin content or an increased capillary density in these regions.

## Author Contributions

E.S. and N.A.A. had access to all data in the study and took responsibility for the integrity of the data and the accuracy of the data analysis. E.S., F.A.U.F., A.K., M.M.B.B., and N.A.A. designed the study. E.S. and F.A.U.F. performed the statistical analysis. N.A.A. supervised the data analysis. A.K., S.E., and W.Z. took responsibility for all steps of the imaging data curation and visualization. X.Y. validated the reproducibility of the data. E.S. wrote the manuscript with the support of N.A.A. All authors critically reviewed and revised the manuscript. E.S. and N.A.A. had primary responsibility for the final content. All authors approved the final draft for submission for publication.

## Funding

This work was supported by the Rhineland Study at the DZNE is predominantly funded by the Federal Ministry of Research, Technology and Space (BMFTR) and the Ministry of Culture and Science of the German State of North Rhine‐Westphalia. N. Ahmad Aziz is partly supported by a European Research Council grant (#101041677). Alexandra Koch is supported by a research grant from the Alzheimer Forschung Initiative e.V. (#25081E). Xingwang Yang is supported by a scholarship from the China Scholarship Council (CSC202108080131).

## Conflicts of Interest

The authors declare no conflicts of interest.

## Supporting information


**Table S1:** Descriptive statistics by physical activity.
**Table S2:** Descriptive statistics by inclusion.
**Table S3:** Minimal differences between analyses in total white matter (WM) and normal appearing white matter (NAWM).
**Table S4:** Orientation dispersion index (ODI) across tracts.
**Table S5:** Beta‐estimates of the association between orientation dispersion and MET hours in significant tracts.
**Table S6:** Mediation results.
**Figure S1:** Flowchart.
**Figure S2:** Flowchart for sensitivity analyses.
**Figure S3:** Effects of physical activity on global macrostructure.
**Figure S4:** Effects of physical activity on global microstructure.
**Figure S5:** Effects of physical activity on regional microstructure (Model 1).
**Figure S6:** Effects of physical activity on regional microstructure (Model 2).
**Figure S7:** Effects of physical activity on regional microstructure (Model 3).
**Figure S8:** Effects of physical activity on regional microstructure (Model 4).
**Figure S9:** Nominally significant interaction effects of metabolic equivalents (MET hours) × age on regional neurite density index (NDI).
**Figure S10:** Nominally significant interaction effects of metabolic equivalents (MET hours) × age on regional mean diffusivity (MD).
**Figure S11:** Age effects vs. effects of energy expenditure on neurite density index (NDI).
**Figure S12:** Effects of metabolic equivalents (MET hours) on global macrostructure.
**Figure S13:** Effects of physical activity on global microstructure.
**Figure S14:** Effects of metabolic equivalents (MET hours) on regional microstructure (Model 1).
**Figure S15:** Effects of metabolic equivalents (MET hours) on regional microstructure (Model 2).
**Figure S16:** Effects of metabolic equivalents (MET hours) on regional microstructure (Model 3).
**Figure S17:** Effects of metabolic equivalents (MET hours) on regional microstructure (Model 4).
**Figure S18:** Analyses in total white matter.
**Figure S19:** Associations between orientation dispersion index (ODI) and C‐reactive protein (CRP).
**Figure S20:** Contributions of variables to cardiometabolic risk components.

**Data S2:** Aging cell author checklist.

## Data Availability

Analytical code will be made available upon request. The data set of the Rhineland Study is not publicly available because of data protection regulations. Access to data can be provided to scientists in accordance with the Rhineland Study's Data Use and Access Policy. Requests for further information or to access the data set of the Rhineland Study should be directed to rs-duac@dzne.de.
