# Supplementary material for: Effects of Physical Activity on Brain White Matter Integrity Are Region‐, Dose‐, and Age‐Dependent: Findings From the Population‐Based Rhineland Study
Source: Aging Cell. 2026 Aug 7;25(8):e70659. doi: 10.1111/acel.70659 (PMC13451344; doi:10.1111/acel.70659)
Supplement: Supplementary file 1 [file ACEL-25-e70659-s001.pdf]

AGING CELL AUTHOR CHECKLIST. ***Authors should submit this checklist together with their manuscript. Please ensure that you have read the Author Guidelines in detail before submission.***

**Title**	Effects of Physical Activity on Brain White Matter Integrity Are Region-, Dose-, and Age-Dependent: Findings From the Population-Based Rhineland Study
**Authors**	Eva Saxler, Fabienne A.U. Fox, Alexandra Koch, Xingwang Yang, Weiyi Zeng, Santiago Estrada, Monique M.B. Breteler, N. Ahmad Aziz
**Manuscript Type**	research article
**Total Character Count (including spaces)[1]**	39,128
**Word count of Summary[2]**	250
**Number of papers cited in the References[3]**	50
**Listing of all Tables (Table1, Table 2 etc)[4]**	Table 1: Sample characteristics

**Figure specifications (please complete one row per figure)[5]** *Figure no.*	Colour *(yes/no)*	Greyscale *(yes/no)*	Black and white *(yes/no)*	Single column (80mm) *(yes/no)*	Double column (180mm) *(yes/no)*	Size of figure at full scale (mm x mm) *(insert details)*	Smallest font size used in the figure at full scale (minimum 6pt) *(insert details)*
1	yes	no	no	yes	no	1805 x 1017	
2	yes	no	no	no	yes	1696 x 1036	
3	yes	no	no	no	yes	1690 x 1016	
4	yes	no	no	no	yes	A: 1117 x 658<br>B: 1117 x 658	
5	yes	no	no	no	yes	806 x 1514	

[1] The maximum character count allowed is 50,000 (incl. spaces) for Primary Research Papers and Reviews, 10,000 for Short Takes.

[2] Summary should not exceed 250 words.

[3] Primary Research Papers can contain a maximum of two tables. If more are needed they should replace some of the Figures or can be placed in the Supporting Information.

[4] A maximum of 45 references is allowed for Primary Research Papers and 20 references for Short Takes.

[5] A Primary Research Paper may contain up to 6 figures and a Short Take up to 2 figures. Authors are encouraged to provide figures in the size they are to appear in the journal and at the specifications given.
