# Supplementary material for: Effects of Physical Activity on Brain White Matter Integrity Are Region‐, Dose‐, and Age‐Dependent: Findings From the Population‐Based Rhineland Study
Source: Aging Cell. 2026 Aug 7;25(8):e70659. doi: 10.1111/acel.70659 (PMC13451344; doi:10.1111/acel.70659)
Supplement: Supplementary file 2 [file ACEL-25-e70659-s002.docx]

**Supplementary Material**

**Table of contents**

[Supplementary Table 1: Descriptive statistics by physical activity	1](#_Toc233895401)

[Supplementary Table 2: Descriptive statistics by inclusion	4](#_Toc233895402)

[Supplementary Table 3: Minimal differences between analyses in total white matter (WM) and normal appearing white matter (NAWM)	6](#_Toc233895403)

[Supplementary Table 4: Orientation dispersion index (ODI) across tracts	7](#_Toc233895404)

[Supplementary Table 5: Beta-estimates of the association between orientation dispersion and MET hours in significant tracts	8](#_Toc233895405)

[Supplementary Table 6: Mediation results	9](#_Toc233895406)

[Supplementary Figure 1: Flowchart	11](#_Toc233895407)

[Supplementary Figure 2: Flowchart for sensitivity analyses	12](#_Toc233895408)

[Analyses with all physical activity measures on global macro- and microstructure	13](#_Toc233895409)

[Supplementary Figure 3: Effects of physical activity on global macrostructure	14](#_Toc233895410)

[Supplementary Figure 4: Effects of physical activity on global microstructure	15](#_Toc233895411)

[Supplementary Figure 5: Effects of physical activity on regional microstructure (Model 1)	16](#_Toc233895412)

[Supplementary Figure 6: Effects of physical activity on regional microstructure (Model 2)	17](#_Toc233895413)

[Supplementary Figure 7: Effects of physical activity on regional microstructure (Model 3)	18](#_Toc233895414)

[Supplementary Figure 8: Effects of physical activity on regional microstructure (Model 4)	19](#_Toc233895415)

[Supplementary Figure 9: Nominally significant interaction effects of metabolic equivalents (MET hours) × age on regional neurite density index (NDI)	20](#_Toc233895416)

[Supplementary Figure 10: Nominally significant interaction effects of metabolic equivalents (MET hours) × age on regional mean diffusivity (MD)	22](#_Toc233895417)

[Supplementary Figure 11: Age effects vs. effects of energy expenditure on neurite density index (NDI)	24](#_Toc233895418)

[Plots from sensitivity analyses	26](#_Toc233895419)

[Supplementary Figure 12: Effects of metabolic equivalents (MET hours) on global macrostructure	27](#_Toc233895420)

[Supplementary Figure 13: Effects of physical activity on global microstructure	28](#_Toc233895421)

[Supplementary Figure 14: Effects of metabolic equivalents (MET hours) on regional microstructure (Model 1)	29](#_Toc233895422)

[Supplementary Figure 15: Effects of metabolic equivalents (MET hours) on regional microstructure (Model 2)	30](#_Toc233895423)

[Supplementary Figure 16: Effects of metabolic equivalents (MET hours) on regional microstructure (Model 3)	31](#_Toc233895424)

[Supplementary Figure 17: Effects of metabolic equivalents (MET hours) on regional microstructure (Model 4)	32](#_Toc233895425)

[Additional analyses	33](#_Toc233895426)

[Supplementary Figure 18: Analyses in total white matter	34](#_Toc233895427)

[Supplementary Figure 19: Associations between orientation dispersion index (ODI) and C-reactive protein (CRP)	35](#_Toc233895428)

[Supplementary Figure 20: Contributions of variables to cardiometabolic risk components	36](#_Toc233895429)

## Supplementary Table 1: Descriptive statistics by physical activity

|  | **N** | **Overall**, N = 4,188 | **Active**, N = 2,094 | **Inactive**, N = 2,094 | **p-value** |
| --- | --- | --- | --- | --- | --- |
| Age (Years) | 4,188 | 55.37 (13.42) | 53.876 (12.33) | 56.862 (14.28) | <0.001 |
| Age Groups | 4,188 |  |  |  | <0.001 |
| 30-39 |  | 665 (16%) | 331 (16%) | 334 (16%) |  |
| 40-49 |  | 690 (16%) | 387 (18%) | 303 (14%) |  |
| 50-59 |  | 1,199 (29%) | 680 (32%) | 519 (25%) |  |
| 60-69 |  | 981 (23%) | 472 (23%) | 509 (24%) |  |
| 70-79 |  | 502 (12%) | 189 (9.0%) | 313 (15%) |  |
| 80-89 |  | 147 (3.5%) | 34 (1.6%) | 113 (5.4%) |  |
| 90+ |  | 4 (<0.1%) | 1 (<0.1%) | 3 (0.1%) |  |
| Sex | 4,188 |  |  |  | <0.001 |
| Male |  | 1,777 (42%) | 820 (39%) | 957 (46%) |  |
| Female |  | 2,411 (58%) | 1,274 (61%) | 1,137 (54%) |  |
| Education Level (ISCED11) | 4,188 |  |  |  | 0.025 |
| Low |  | 68 (1.6%) | 31 (1.5%) | 37 (1.8%) |  |
| Middle |  | 1,843 (44%) | 881 (42%) | 962 (46%) |  |
| High |  | 2,277 (54%) | 1,182 (56%) | 1,095 (52%) |  |
| Body Mass Index (kg/m²) | 4,177 | 25.60 (4.14) | 24.89 (3.70) | 26.30 (4.42) | <0.001 |
| Missing |  | 11 | 4 | 7 |  |
| Current smoker | 4,183 |  |  |  | <0.001 |
| No |  | 3,663 (88%) | 1,872 (90%) | 1,791 (86%) |  |
| Yes |  | 520 (12%) | 218 (10%) | 302 (14%) |  |
| Missing |  | 5 | 4 | 1 |  |
| Hypertension (binary) | 4,147 |  |  |  | <0.001 |
| No |  | 2,707 (65%) | 1,482 (72%) | 1,225 (59%) |  |
| Yes |  | 1,440 (35%) | 588 (28%) | 852 (41%) |  |
| Missing |  | 41 | 24 | 17 |  |
| Systolic Blood Pressure (mmHg) | 4,165 | 125.73 (15.516) | 124.03 (15.10) | 127.44 (15.74) | <0.001 |
| Missing |  | 23 | 10 | 13 |  |
| Diastolic Blood Pressure (mmHg) | 4,165 | 74.65 (9.12) | 74.08 (9.12) | 75.22 (9.08) | <0.001 |
| Missing |  | 23 | 10 | 13 |  |
| Neurological Disorders (binary) | 4,188 |  |  |  | 0.009 |
| No |  | 4,078 (97%) | 2,053 (98%) | 2,025 (97%) |  |
| Yes |  | 110 (2.6%) | 41 (2.0%) | 69 (3.3%) |  |
| Actimetry sensor: sum of hours worn | 4,188 | 166.87 (4.54) | 167.40 (3.32) | 166.35 (5.46) | <0.001 |
| Average weighted daily MET hours | 4,188 | 34.02 (1.34) | 35.06 (0.97) | 32.98 (0.70) | <0.001 |
| Average weighted daily step count | 4,188 | 8,844.66 (3,217.31) | 11,228.89 (2,550.21) | 6,460.44 (1,681.97) | <0.001 |
| Average weighted daily % light intensity PA | 4,188 | 0.21 (0.06) | 0.23 (0.06) | 0.19 (0.06) | <0.001 |
| Average weighted daily % MVPA | 4,188 | 0.05 (0.02) | 0.06 (0.01) | 0.03 (0.01) | <0.001 |
| Average weighted daily % sedentary PA | 4,188 | 0.74 (0.07) | 0.71 (0.06) | 0.78 (0.06) | <0.001 |
| Total brain volume (cm³) | 4,165 | 1,104,480.49 (116,055.71) | 1,109,130.40 (113,721.30) | 1,099,823.88 (118,191.41) | 0.010 |
| Missing |  | 23 | 10 | 13 |  |
| Total white matter volume (cm³) | 4,174 | 455,311.83 (57,831.08) | 456,886.62 (56,861.06) | 453,735.53 (58,757.38) | 0.078 |
| Missing |  | 14 | 6 | 8 |  |
| Estimated total intracranial volume (cm³) | 4,175 | 1,549,909.76 (150,816.04) | 1,549,065.49 (145,991.79) | 1,550,754.43 (155,523.37) | 0.7 |
| Missing |  | 13 | 6 | 7 |  |
| Total volume of supratentorial WMH (cm³) | 4,188 | 1.73 (4.18) | 1.36 (3.13) | 2.10 (4.99) | <0.001 |
| Global mean FA in skeletonized NAWM | 4,188 | 0.62 (0.02) | 0.62 (0.02) | 0.62 (0.02) | <0.001 |
| Global mean MD in skeletonized NAWM * e10 | 4,188 | 7.41 (0.28) | 7.39 (0.26) | 7.44 (0.30) | <0.001 |
| Global mean ODI in skeletonized NAWM | 4,188 | 0.22 (0.01) | 0.22 (0.01) | 0.22 (0.01) | <0.001 |
| Global mean NDI in skeletonized NAWM | 4,188 | 0.67 (0.03) | 0.67 (0.03) | 0.67 (0.04) | 0.061 |
| Global mean ISOVF in skeletonized NAWM | 4,188 | 0.04 (0.01) | 0.04 (0.01) | 0.04 (0.01) | <0.001 |
| Abbreviations: ISCED = International Standard Classification of Education; ISOVF = isotropic volume fraction; MET = metabolic equvalents; PA = physical activity; MVPA = moderate-to-vigorous PA; WMH = white matter hyperintensities; FA = fractional anisotropy; MD = mean diffusivity; ODI = orientation dispersion index; NDI = neurite density index; NAWM = normal-appearing white matter  Categorical data: n (%); continuous data: mean (SD)  Note. To assess group differences between active and inactive participants, a χ2 test was used for categorical variables and a 2-sample t-test for continuous variables. | | | | | |

## Supplementary Table 2: Descriptive statistics by inclusion

|  | **N** | **Overall**, N = 8,314 | **Excluded**, N = 4,126 | **Included**, N = 4,188 | **p-value** |
| --- | --- | --- | --- | --- | --- |
| Age (Years) | 8,314 | 55.88 (13.84) | 56.39 (14.23) | 55.37 (13.42) | <0.001 |
| Age Groups | 8,314 |  |  |  | <0.001 |
| 30-39 |  | 1,261 (15%) | 596 (14%) | 665 (16%) |  |
| 40-49 |  | 1,443 (17%) | 753 (18%) | 690 (16%) |  |
| 50-59 |  | 2,291 (28%) | 1,092 (26%) | 1,199 (29%) |  |
| 60-69 |  | 1,821 (22%) | 840 (20%) | 981 (23%) |  |
| 70-79 |  | 1,094 (13%) | 592 (14%) | 502 (12%) |  |
| 80-89 |  | 388 (4.7%) | 241 (5.8%) | 147 (3.5%) |  |
| 90+ |  | 16 (0.2%) | 12 (0.3%) | 4 (<0.1%) |  |
| Sex | 8,314 |  |  |  | 0.036 |
| Male |  | 3,623 (44%) | 1,846 (45%) | 1,777 (42%) |  |
| Female |  | 4,691 (56%) | 2,280 (55%) | 2,411 (58%) |  |
| Education Level (ISCED11) | 8,210 |  |  |  | 0.001 |
| Low |  | 162 (2.0%) | 94 (2.3%) | 68 (1.6%) |  |
| Middle |  | 3,725 (45%) | 1,882 (47%) | 1,843 (44%) |  |
| High |  | 4,323 (53%) | 2,046 (51%) | 2,277 (54%) |  |
| Missing |  | 104 | 104 | 0 |  |
| Body Mass Index (kg/m²) | 8,161 | 26.04 (4.65) | 26.51 (5.10) | 25.60 (4.14) | <0.001 |
| Missing |  | 153 | 142 | 11 |  |
| Current smoker | 8,207 |  |  |  | 0.5 |
| No |  | 7,207 (88%) | 3,544 (88%) | 3,663 (88%) |  |
| Yes |  | 1,000 (12%) | 480 (12%) | 520 (12%) |  |
| Missing |  | 107 | 102 | 5 |  |
| Hypertension (binary) | 8,212 |  |  |  | <0.001 |
| No |  | 5,125 (62%) | 2,418 (59%) | 2,707 (65%) |  |
| Yes |  | 3,087 (38%) | 1,647 (41%) | 1,440 (35%) |  |
| Missing |  | 102 | 61 | 41 |  |
| Systolic Blood Pressure (mmHg) | 8,253 | 126.38 (15.88) | 127.03 (16.22) | 125.73 (15.52) | <0.001 |
| Missing |  | 61 | 38 | 23 |  |
| Diastolic Blood Pressure (mmHg) | 8,253 | 74.94 (9.19) | 75.24 (9.25) | 74.65 (9.12) | 0.004 |
| Missing |  | 61 | 38 | 23 |  |
| Neurological Disorders (binary) | 8,314 |  |  |  | <0.001 |
| No |  | 7,979 (96%) | 3,901 (95%) | 4,078 (97%) |  |
| Yes |  | 335 (4.0%) | 225 (5.5%) | 110 (2.6%) |  |
| Actimetry sensor: sum of hours worn | 6,441 | 163.46 (20.21) | 157.12 (32.67) | 166.87 (4.54) | <0.001 |
| Missing |  | 1,873 | 1,873 | 0 |  |
| Average weighted daily MET hours | 6,160 | 33.97 (1.38) | 33.87 (1.44) | 34.02 (1.34) | <0.001 |
| Missing |  | 2,154 | 2,154 | 0 |  |
| Average weighted daily step count | 6,160 | 8,741.05 (3,284.23) | 8,521.01 (3,412.44) | 8,844.66 (3,217.31) | <0.001 |
| Missing |  | 2,154 | 2,154 | 0 |  |
| Average weighted daily % light intensity PA | 6,160 | 0.21 (0.06) | 0.21 (0.06) | 0.21 (0.06) | 0.026 |
| Missing |  | 2,154 | 2,154 | 0 |  |
| Average weighted daily % MVPA | 6,160 | 0.05 (0.02) | 0.05 (0.02) | 0.05 (0.02) | <0.001 |
| Missing |  | 2,154 | 2,154 | 0 |  |
| Average weighted daily % sedentary PA | 6,160 | 0.74 (0.07) | 0.75 (0.07) | 0.74 (0.07) | 0.004 |
| Missing |  | 2,154 | 2,154 | 0 |  |
| Total brain volume (cm³) | 5,839 | 1,103,485.29 (117,753.19) | 1,101,009.18 (121,874.24) | 1,104,480.49 (116,055.71) | 0.3 |
| Missing |  | 2,475 | 2,452 | 23 |  |
| Total white matter volume (cm³) | 5,855 | 454,910.49 (58,846.85) | 453,913.93 (61,302.61) | 455,311.83 (57,831.08) | 0.4 |
| Missing |  | 2,459 | 2,445 | 14 |  |
| Estimated total intracranial volume (cm³) | 5,892 | 1,550,086.96 (152,075.12) | 1,550,517.83 (155,137.64) | 1,549,909.76 (150,816.04) | 0.9 |
| Missing |  | 2,422 | 2,409 | 13 |  |
| Total volume of supratentorial WMH (cm³) | 5,747 | 1.80 (4.27) | 1.97 (4.51) | 1.73 (4.18) | 0.074 |
| Missing |  | 2,567 | 2,567 | 0 |  |
| Global mean FA in skeletonized NAWM | 5,546 | 0.62 (0.02) | 0.62 (0.02) | 0.62 (0.02) | 0.6 |
| Missing |  | 2,768 | 2,768 | 0 |  |
| Global mean MD in skeletonized NAWM * e10 | 5,546 | 7.41 (0.29) | 7.40 (0.29) | 7.41 (0.28) | 0.066 |
| Missing |  | 2,768 | 2,768 | 0 |  |
| Global mean ODI in skeletonized NAWM | 5,546 | 0.22 (0.01) | 0.22 (0.01) | 0.22 (0.01) | 0.4 |
| Missing |  | 2,768 | 2,768 | 0 |  |
| Global mean NDI in skeletonized NAWM | 5,546 | 0.67 (0.03) | 0.67 (0.03) | 0.67 (0.03) | 0.7 |
| Missing |  | 2,768 | 2,768 | 0 |  |
| Global mean ISOVF in skeletonized NAWM | 5,546 | 0.04 (0.01) | 0.04 (0.01) | 0.04 (0.01) | 0.006 |
| Missing |  | 2,768 | 2,768 | 0 |  |
| Abbreviations: ISCED = International Standard Classification of Education; ISOVF = isotropic volume fraction; MET = metabolic equvalents; PA = physical activity; MVPA = moderate-to-vigorous PA; WMH = white matter hyperintensities; FA = fractional anisotropy; MD = mean diffusivity; ODI = orientation dispersion index; NDI = neurite density index; NAWM = normal-appearing white matter  Categorical data: n (%); continuous data: mean (SD)  Note. To assess group differences between included and excluded participants, a χ2 test was used for categorical variables and a 2-sample t test for continuous variables. | | | | | |

## Supplementary Table 3: Minimal differences between analyses in total white matter (WM) and normal appearing white matter (NAWM)

**Tract	MRI measure	Beta estimate	p-value	Model**

PCR	MD	-0.036	0.013	NAWM

PCR	MD	-0.026	0.054	WM

SCR	MD	-0.045	0.001	NAWM

SCR	MD	-0.041	0.002	WM

SFOF	FA	0.028	0.072	NAWM

SFOF	FA	0.030	0.045	WM

**Note**. Beta-estimates are displayed for MET hours in Model 3.

**Abbreviations**: FA: fractional anisotropy; MD: mean diffusivity; SCR: superior corona radiata; SFOF: superior fronto-occipital fasciculus; PCR: posterior corona radiata.

## Supplementary Table 4: Orientation dispersion index (ODI) across tracts

**No.	tract	mean ODI**

1	PCT	0.303

2	CH	0.263

3	CST	0.259

4	ACR	0.258

5	ML	0.254

6	SCR	0.244

7	FST	0.242

8	MCP	0.235

9	ICP	0.229

10	EC	0.228

11	PCR	0.215

12	CP	0.208

13	ALIC	0.206

14	SFOF	0.201

15	UF	0.194

16	SLF	0.189

17	RIC	0.188

18	PLIC	0.181

19	SS	0.171

20	FOR	0.161

21	CCG	0.153

22	GCC	0.149

23	BCC	0.133

24	PTR	0.129

25	TAP	0.128

26	SCC	0.126

27	SCP	0.112

**Note**. Mean ODI in 27 tracts, sorted from largest to smallest.

**Abbreviations:** ACR = anterior corona radiata; ALIC = anterior limb of internal capsule; BCC = body of corpus callosum; CCG = cingulum /cingulate gyrus; CH = cingulum hippocampus; CP = cerebral peduncle; CST = corticospinal tract; EC = external capsule; FOR = fornix; FST = fornix / stria terminalis; GCC = genu of corpus callosum; ICP = inferior cerebellar peduncle; MCP = middle cerebellar peduncle; ML = medial lemniscus; PCR = posterior corona radiata; PCT = pontine crossing tract; PLIC = posterior limb of internal capsule; PTR = posterior thalamic radiation; RIC = rostral limb of internal capsule; SCC = splenium of corpus callosum; SCP = superior cerebellar peduncle; SCR = superior corona radiata; SFOF = superior fronto-occipital fasciculus; SLF = superior longitudinal fasciculus; SS = sagittal striatum; TAP = tapetum; UF = uncinate fasciculus.

## Supplementary Table 5: Beta-estimates of the association between orientation dispersion and MET hours in significant tracts

**No.	Tract	Beta estimate	p-value**

1	SCC	-0.121	0.000

2	CP	-0.119	0.000

3	MCP	-0.117	0.000

4	SCP	-0.115	0.000

5	PLIC	-0.110	0.000

6	CH	-0.092	0.000

7	BCC	-0.091	0.000

8	CST	-0.089	0.000

9	ICP	-0.088	0.000

10	FST	-0.082	0.000

11	PTR	-0.078	0.000

12	RIC	-0.076	0.000

13	PCT	-0.072	0.000

14	PCR	-0.068	0.000

15	SS	-0.064	0.000

16	TAP	-0.056	0.000

17	ALIC	-0.053	0.001

18	ML	-0.052	0.001

19	SLF	-0.050	0.001

20	GCC	-0.047	0.003

21	CCG	-0.043	0.005

22	FOR	-0.043	0.002

23	EC	-0.040	0.009

24	UF	-0.036	0.020

25	SFOF	-0.035	0.025

26	ACR	-0.033	0.029

**Note**. Significant (p < 0.05) beta-estimates, printed from largest to smallest absolute value.

**Abbreviations:** ACR = anterior corona radiata; ALIC = anterior limb of internal capsule; BCC = body of corpus callosum; CCG = cingulum /cingulate gyrus; CH = cingulum hippocampus; CP = cerebral peduncle; CST = corticospinal tract; EC = external capsule; FOR = fornix; FST = fornix / stria terminalis; GCC = genu of corpus callosum; ICP = inferior cerebellar peduncle; MCP = middle cerebellar peduncle; ML = medial lemniscus; PCR = posterior corona radiata; PCT = pontine crossing tract; PLIC = posterior limb of internal capsule; PTR = posterior thalamic radiation; RIC = rostral limb of internal capsule; SCC = splenium of corpus callosum; SCP = superior cerebellar peduncle; SCR = superior corona radiata; SFOF = superior fronto-occipital fasciculus; SLF = superior longitudinal fasciculus; SS = sagittal striatum; TAP = tapetum; UF = uncinate fasciculus.

## Supplementary Table 6: Mediation results

| Mediation method | Mediator | Outcome | Indirect | | | Direct | | | Total | | | Percentage mediated | | |
| --- | --- | --- | --- | --- | --- | --- | --- | --- | --- | --- | --- | --- | --- | --- |
|  |  |  | **-1 SD** | **Average** | **+1 SD** | **-1 SD** | **Average** | **+1 SD** | **-1 SD** | **Average** | **+1 SD** | **-1 SD** | **Average** | **+1 SD** |
| Single variable | BMI | Global FA | 0.01 [0.002; 0.019]* | 0.008 [0.001; 0.014]* | 0.006 [0.001; 0.011]* | - | 0.027 [-0.005; 0.054] | - | 0.037 [0.007; 0.063]* | 0.035 [0.004; 0.061]* | 0.033 [0.002; 0.058]* | 0.274 | 0.230 | 0.180 |
| Single variable | BMI | Global NDI | -0.048 [-0.062; -0.035]*** | -0.038 [-0.047; -0.029]*** | -0.028 [-0.036; -0.021]*** | - | -0.007 [-0.038; 0.021] | - | -0.055 [-0.089; -0.026]** | -0.045 [-0.077; -0.016]** | -0.035 [-0.067; -0.008]* | 0.871 | 0.843 | 0.798 |
| Single variable | BMI | Global ODI | -0.101 [-0.126; -0.077]*** | -0.08 [-0.096; -0.066]*** | -0.059 [-0.072; -0.046]*** | -0.121 [-0.173; -0.069]*** | -0.072 [-0.102; -0.041]*** | -0.024 [-0.06; 0.008] | -0.222 [-0.279; -0.164]*** | -0.152 [-0.185; -0.12]*** | -0.083 [-0.118; -0.048]*** | 0.454 | 0.524 | 0.710 |
| Single variable | BMI | Global ISOVF | -0.08 [-0.102; -0.062]*** | -0.063 [-0.077; -0.052]*** | -0.047 [-0.058; -0.036]*** | - | -0.026 [-0.06; 0.005] | - | -0.106 [-0.142; -0.069]*** | -0.089 [-0.123; -0.059]*** | -0.072 [-0.107; -0.044]*** | 0.756 | 0.710 | 0.643 |
| Single variable | BP | Global FA | - | 0.003 [0.001; 0.006]* | - | 0.077 [0.02; 0.126]** | 0.041 [0.008; 0.069]** | 0.005 [-0.034; 0.042] | 0.08 [0.025; 0.13]** | 0.044 [0.011; 0.072]** | 0.008 [-0.031; 0.045] | 0.039 | 0.072 | 0.410 |
| Single variable | BP | Global NDI | - | -0.001 [-0.003; 0.001] | - | - | -0.044 [-0.076; -0.015]** | - | - | -0.044 [-0.076; -0.015]** | - | - | 0.015 | - |
| Single variable | BP | Global ODI | - | -0.006 [-0.011; -0.003]** | - | -0.222 [-0.277; -0.169]*** | -0.15 [-0.181; -0.119]*** | -0.078 [-0.115; -0.045]*** | -0.229 [-0.284; -0.174]*** | -0.156 [-0.187; -0.125]*** | -0.084 [-0.121; -0.051]*** | 0.028 | 0.041 | 0.076 |
| Single variable | BP | Global ISOVF | - | -0.004 [-0.007; -0.001]* | - | - | -0.083 [-0.116; -0.051]*** | - | - | -0.087 [-0.119; -0.055]*** | - | - | 0.042 | - |
| Single variable | CRP | Global FA | 0.001 [-0.005; 0.008] | 0.001 [-0.004; 0.006] | 0.001 [-0.002; 0.004] | 0.081 [0.024; 0.134]** | 0.046 [0.015; 0.078]** | 0.011 [-0.026; 0.048] | 0.083 [0.028; 0.135]** | 0.047 [0.017; 0.079]** | 0.012 [-0.026; 0.048] | 0.017 | 0.022 | 0.056 |
| Single variable | CRP | Global NDI | -0.016 [-0.025; -0.009]*** | -0.012 [-0.018; -0.007]*** | -0.008 [-0.013; -0.004]** | - | -0.03 [-0.058; 0.001]* | - | -0.047 [-0.076; -0.016]** | -0.042 [-0.072; -0.012]** | -0.038 [-0.067; -0.008]* | 0.350 | 0.284 | 0.203 |
| Single variable | CRP | Global ODI | -0.033 [-0.045; -0.024]*** | -0.025 [-0.033; -0.018]*** | -0.016 [-0.022; -0.01]*** | -0.194 [-0.255; -0.141]*** | -0.131 [-0.165; -0.1]*** | -0.068 [-0.104; -0.036]*** | -0.228 [-0.288; -0.17]*** | -0.156 [-0.191; -0.125]*** | -0.084 [-0.118; -0.052]*** | 0.146 | 0.158 | 0.187 |
| Single variable | CRP | Global ISOVF | -0.026 [-0.038; -0.017]*** | -0.019 [-0.027; -0.013]*** | -0.012 [-0.019; -0.008]*** | - | -0.066 [-0.097; -0.032]*** | - | -0.093 [-0.123; -0.06]*** | -0.086 [-0.115; -0.053]*** | -0.079 [-0.108; -0.044]*** | 0.284 | 0.226 | 0.158 |
| FAMD | Component 1 | Global FA | - | 0.006 [0.001; 0.011]* | - | - | 0.032 [0; 0.061]* | - | - | 0.037 [0.007; 0.067]* | - | - | 0.153 | - |
| FAMD | Component 1 | Global NDI | - | -0.015 [-0.021; -0.009]*** | - | - | -0.023 [-0.053; 0.007] | - | - | -0.038 [-0.067; -0.007]* | - | - | 0.393 | - |
| FAMD | Component 1 | Global ODI | - | -0.034 [-0.043; -0.026]*** | - | -0.192 [-0.247; -0.14]*** | -0.121 [-0.152; -0.09]*** | -0.049 [-0.084; -0.019]** | -0.226 [-0.281; -0.172]*** | -0.154 [-0.186; -0.123]*** | -0.083 [-0.117; -0.051]*** | 0.149 | 0.218 | 0.405 |
| FAMD | Component 1 | Global ISOVF | - | -0.03 [-0.038; -0.023]*** | - | - | -0.05 [-0.079; -0.014]** | - | - | -0.08 [-0.111; -0.044]*** | - | - | 0.375 | - |
| FAMD | Component 2 | Global FA | - | 0.002 [-0.001; 0.005] | - | - | 0.036 [0.007; 0.066]* | - | - | 0.037 [0.007; 0.067]* | - | - | 0.044 | - |
| FAMD | Component 2 | Global NDI | - | -0.005 [-0.009; -0.002]** | - | - | -0.033 [-0.062; -0.001]* | - | - | -0.038 [-0.067; -0.007]* | - | - | 0.137 | - |
| FAMD | Component 2 | Global ODI | - | -0.012 [-0.017; -0.008]*** | - | -0.21 [-0.266; -0.158]*** | -0.141 [-0.175; -0.11]*** | -0.072 [-0.107; -0.039]*** | -0.223 [-0.279; -0.169]*** | -0.154 [-0.187; -0.121]*** | -0.084 [-0.117; -0.049]*** | 0.055 | 0.080 | 0.146 |
| FAMD | Component 2 | Global ISOVF | - | -0.009 [-0.014; -0.005]*** | - | - | -0.071 [-0.102; -0.035]*** | - | - | -0.08 [-0.111; -0.044]*** | - | - | 0.107 | - |

**Abbreviations**: BMI = body mass index; BP = blood pressure; CRP = C-reactive protein; FA = fractional anisotropy; FAMD = factor analysis for mixed data; ISOVF = isotropic volume fraction; MD = mean diffusivity; NDI = neurite density index; ODI = orientation dispersion index.

**Note**: * p < 0.05; ** p < 0.01; *** p < 0.0001

Where there is no estimate provided, quadratic terms did not reach statistical significance and were removed.

Predictor was always energy expenditure (metabolic equivalents).

## Supplementary Figure 1: Flowchart

n = 1930: missing dMRI data

n = 42: missing covariate data (education)

Rhineland Study Participants

n = 8314

Participants with valid actimetry recordings

n = 6160

Analytical sample

n = 4188

n = 1936: missing actimetry data

n = 218: incomplete actimetry data

TAP:

n = 4159

SFOF:

n = 4185

FOR:

n = 4176

Model 4: n = 4131

Model 4: n = 4105

Model 4: n = 4122

n = 54: missing cardiometabolic data

Model 4:

n= 4134

**Abbreviations:** dMRI = diffusion-weighted MRI; FOR = fornix; SFOF = superior fronto-occipital fasciculus.

## Supplementary Figure 2: Flowchart for sensitivity analyses

Main sample

n = 4188

Participants with no outliers in METs, global mean diffusion data, TBV, WMV, WMHv, or covariates

n = 3912

Participants with no neurological illnesses or events

n = 4078

Model 4

n = 3858

SFOF

n = 3910

TAP

n = 3896

FOR

n = 3900

Model 4

n = 3856

Model 4

n = 3842

Model 4

n = 3846

Missing data in individual tracts

**Abbreviations:** FOR = fornix; METs = metabolic equivalents; SFOF = superior fronto-occipital fasciculus; TAP = tapetum; TBV = total brain volume; WMHv = total volume of white matter hyperintensities; WMV = white matter volume.

# Analyses with all physical activity measures on global macro- and microstructure

## Supplementary Figure 3: Effects of physical activity on global macrostructure

**Note:** * p < 0.05; ** p < 0.01

Analyses were based on a sample of n = 4164, as 23 participants had missing MRI-data for these variables and one had an erroneous value in estimated total intracranial volume. In Model 4, additional n = 53 participants with missing data were excluded.

For all three macrostructural outcomes, we additionally controlled for estimated intracranial volume. Higher order terms (i.e. quadratic and interaction terms) were only re-entered in consecutive models if they reached statistical significance.

**Abbreviations**: MET = metabolic equivalents; MVPA = moderate-to-vigorous physical activity; TBV = total brain volume; WMV = white matter volume; WMHv = total volume of white matter hyperintensities.

## Supplementary Figure 4: Effects of physical activity on global microstructure

**Note:** * p < 0.05; ** p < 0.013

Higher order terms (i.e. quadratic and interaction terms) were only re-entered in consecutive models if they reached statistical significance.

**Abbreviations**: FA = fractional anisotropy; ISOVF = isotropic volume fraction; MD = mean diffusivity; MET = metabolic equivalents; MVPA = moderate-to-vigorous physical activity; NDI = neurite density index; ODI = orientation dispersion index.

## Supplementary Figure 5: Effects of physical activity on regional microstructure (Model 1)

**Note:** * p < 0.05; ** p < 0.0008

**Abbreviations:** ACR = anterior corona radiata; ALIC = anterior limb of internal capsule; BCC = body of corpus callosum; CCG = cingulum /cingulate gyrus; CH = cingulum hippocampus; CP = cerebral peduncle; CST = corticospinal tract; EC = external capsule; FA = fractional anisotropy; FOR = fornix; FST = fornix / stria terminalis; GCC = genu of corpus callosum; ICP = inferior cerebellar peduncle; ISOVF = isotropic volume fraction; MCP = middle cerebellar peduncle; MD = mean diffusivity; MET = metabolic equivalents; ML = medial lemniscus; MVPA = moderate-to-vigorous physical activity; NDI = neurite density index; ODI = orientation dispersion index; PCR = posterior corona radiata; PCT = pontine crossing tract; PLIC = posterior limb of internal capsule; PTR = posterior thalamic radiation; RIC = rostral limb of internal capsule; SCC = splenium of corpus callosum; SCP = superior cerebellar peduncle; SCR = superior corona radiata; SFOF = superior fronto-occipital fasciculus; SLF = superior longitudinal fasciculus; SS = sagittal striatum; TAP = tapetum; UF = uncinate fasciculus.

## Supplementary Figure 6: Effects of physical activity on regional microstructure (Model 2)

**Note:** * p < 0.05; ** p < 0.0008

**Abbreviations:** ACR = anterior corona radiata; ALIC = anterior limb of internal capsule; BCC = body of corpus callosum; CCG = cingulum /cingulate gyrus; CH = cingulum hippocampus; CP = cerebral peduncle; CST = corticospinal tract; EC = external capsule; FA = fractional anisotropy; FOR = fornix; FST = fornix / stria terminalis; GCC = genu of corpus callosum; ICP = inferior cerebellar peduncle; ISOVF = isotropic volume fraction; MCP = middle cerebellar peduncle; MD = mean diffusivity; MET = metabolic equivalents; ML = medial lemniscus; MVPA = moderate-to-vigorous physical activity; NDI = neurite density index; ODI = orientation dispersion index; PCR = posterior corona radiata; PCT = pontine crossing tract; PLIC = posterior limb of internal capsule; PTR = posterior thalamic radiation; RIC = rostral limb of internal capsule; SCC = splenium of corpus callosum; SCP = superior cerebellar peduncle; SCR = superior corona radiata; SFOF = superior fronto-occipital fasciculus; SLF = superior longitudinal fasciculus; SS = sagittal striatum; TAP = tapetum; UF = uncinate fasciculus.

## Supplementary Figure 7: Effects of physical activity on regional microstructure (Model 3)

**Note:** * p < 0.05; ** p < 0.0008

**Abbreviations:** ACR = anterior corona radiata; ALIC = anterior limb of internal capsule; BCC = body of corpus callosum; CCG = cingulum /cingulate gyrus; CH = cingulum hippocampus; CP = cerebral peduncle; CST = corticospinal tract; EC = external capsule; FA = fractional anisotropy; FOR = fornix; FST = fornix / stria terminalis; GCC = genu of corpus callosum; ICP = inferior cerebellar peduncle; ISOVF = isotropic volume fraction; MCP = middle cerebellar peduncle; MD = mean diffusivity; MET = metabolic equivalents; ML = medial lemniscus; MVPA = moderate-to-vigorous physical activity; NDI = neurite density index; ODI = orientation dispersion index; PA = physical activity; PCR = posterior corona radiata; PCT = pontine crossing tract; PLIC = posterior limb of internal capsule; PTR = posterior thalamic radiation; RIC = rostral limb of internal capsule; SCC = splenium of corpus callosum; SCP = superior cerebellar peduncle; SCR = superior corona radiata; SFOF = superior fronto-occipital fasciculus; SLF = superior longitudinal fasciculus; SS = sagittal striatum; TAP = tapetum; UF = uncinate fasciculus.

## Supplementary Figure 8: Effects of physical activity on regional microstructure (Model 4)

**Note:** * p < 0.05; ** p < 0.0008

**Abbreviations:** ACR = anterior corona radiata; ALIC = anterior limb of internal capsule; BCC = body of corpus callosum; CCG = cingulum /cingulate gyrus; CH = cingulum hippocampus; CP = cerebral peduncle; CST = corticospinal tract; EC = external capsule; FA = fractional anisotropy; FOR = fornix; FST = fornix / stria terminalis; GCC = genu of corpus callosum; ICP = inferior cerebellar peduncle; ISOVF = isotropic volume fraction; MCP = middle cerebellar peduncle; MD = mean diffusivity; MET = metabolic equivalents; ML = medial lemniscus; MVPA = moderate-to-vigorous physical activity; NDI = neurite density index; ODI = orientation dispersion index; PCR = posterior corona radiata; PCT = pontine crossing tract; PLIC = posterior limb of internal capsule; PTR = posterior thalamic radiation; RIC = rostral limb of internal capsule; SCC = splenium of corpus callosum; SCP = superior cerebellar peduncle; SCR = superior corona radiata; SFOF = superior fronto-occipital fasciculus; SLF = superior longitudinal fasciculus; SS = sagittal striatum; TAP = tapetum; UF = uncinate fasciculus.

## Supplementary Figure 9: Nominally significant interaction effects of metabolic equivalents (MET hours) × age on regional neurite density index (NDI)

**Note.** All plotted effects were nominally significant with p < 0.05. For illustrative purposes, age was split into two groups with a median split; analyses were based on continuous data. All continuous variables were z-standardized.

**Abbreviations:** ACR = anterior corona radiata; BCC = body of corpus callosum; CCG = cingulum cingulate gyrus; GCC = genu of corpus callosum; ICP = inferior cerebellar peduncle; MCP = middle cerebellar peduncle; MET = metabolic equivalents; ML = medial lemniscus; NDI = neurite density index; SCR = superior cerebellar peduncle; SFOF = superior fronto-occipital fasciculus; SLF = superior longitudinal fasciculus; UF = uncinate fasciculus.

## Supplementary Figure 10: Nominally significant interaction effects of metabolic equivalents (MET hours) × age on regional mean diffusivity (MD)

**Note.** All plotted effects were nominally significant with p < 0.05. For illustrative purposes, age was split into two groups with a median split; analyses were based on continuous data. All continuous variables were z-standardized.

**Abbreviations:** BCC = body of corpus callosum; FST = fornix/stria terminalis; GCC = genu of corpus callosum; ICP = inferior cerebellar peduncle; MCP = middle cerebellar peduncle; MD = mean diffusivity; MET = metabolic equivalents; PLIC = posterior limb of internal capsule; RIC = rostral limb of internal capsule; SCC = splenium of corpus callosum; SFOF = superior fronto-occipital fasciculus; TAP = tapetum.

## Supplementary Figure 11: Age effects vs. effects of energy expenditure on neurite density index (NDI)

**Note**. The x axis provides uniform z-scores, as both age and METs were z-standardized. This enables direct comparison of the direction of effects.

**Abbreviations**: CH = cingulum/hippocampus; CP = cerebral peduncle; CST = corticospinal tract; FST = fornix/stria terminalis; ICP = inferior cerebellar peduncle; MCP = middle cerebellar peduncle; MET = metabolic equivalents; ML = medial lemniscus; NDI = neurite density index; PCT = pontine crossing tract; PLIC = posterior limb of internal capsule; SCC = splenium of corpus callosum; SCP = superior cerebellar peduncle.

# Plots from sensitivity analyses

(outliers and participants with neurological diseases excluded)

## Supplementary Figure 12: Effects of metabolic equivalents (MET hours) on global macrostructure

**Note:** * p < 0.05; ** p < 0.01

Analyses were based on a sample of n = 3897, as 15 participants had missing MRI-data for these variables. In Model 4, additional n = 53 participants with missing data were excluded.

For all three macrostructural outcomes, we additionally controlled for estimated intracranial volume. Higher order terms (i.e. quadratic and interaction terms) were only re-entered in consecutive models if they reached statistical significance.

**Abbreviations**: MET = metabolic equivalents; MVPA = moderate-to-vigorous physical activity; TBV = total brain volume; WMV = white matter volume; WMHv = total volume of white matter hyperintensities

## Supplementary Figure 13: Effects of physical activity on global microstructure

**Note:** * p < 0.05; ** p < 0.013

Corrected p-value derived from correlation matrix of five diffusion measures in the sensitivity-dataset.

Higher order terms (i.e. quadratic and interaction terms) were only re-entered in consecutive models if they reached statistical significance.

**Abbreviations**: FA = fractional anisotropy; ISOVF = isotropic volume fraction; MET = metabolic equivalents; MD = mean diffusivity; NDI = neurite density index; ODI = orientation dispersion index.

## Supplementary Figure 14: Effects of metabolic equivalents (MET hours) on regional microstructure (Model 1)

**Note:** * p < 0.05; ** p < 0.0007

Corrected p-value derived from correlation matrix of 27 tracts × five diffusion measures in the sensitivity-dataset.

**Abbreviations:** ACR = anterior corona radiata; ALIC = anterior limb of internal capsule; BCC = body of corpus callosum; CCG = cingulum /cingulate gyrus; CH = cingulum hippocampus; CP = cerebral peduncle; CST = corticospinal tract; dMRI = diffusion-weighted MRI; EC = external capsule; FA = fractional anisotropy; FOR = fornix; FST = fornix / stria terminalis; GCC = genu of corpus callosum; ICP = inferior cerebellar peduncle; ISOVF = isotropic volume fraction; MCP = middle cerebellar peduncle; MD = mean diffusivity; MET = metabolic equivalents; ML = medial lemniscus; NDI = neurite density index; ODI = orientation dispersion index; PCR = posterior corona radiata; PCT = pontine crossing tract; PLIC = posterior limb of internal capsule; PTR = posterior thalamic radiation; RIC = rostral limb of internal capsule; SCC = splenium of corpus callosum; SCP = superior cerebellar peduncle; SCR = superior corona radiata; SFOF = superior fronto-occipital fasciculus; SLF = superior longitudinal fasciculus; SS = sagittal striatum; TAP = tapetum; UF = uncinate fasciculus.

## Supplementary Figure 15: Effects of metabolic equivalents (MET hours) on regional microstructure (Model 2)

**Note:** * p < 0.05; ** p < 0.0007

Corrected p-value derived from correlation matrix of 27 tracts × five diffusion measures in the sensitivity-dataset.

Higher order terms (i.e. quadratic and interaction terms) were only re-entered in consecutive models if they reached statistical significance.

**Abbreviations:** ACR = anterior corona radiata; ALIC = anterior limb of internal capsule; BCC = body of corpus callosum; CCG = cingulum /cingulate gyrus; CH = cingulum hippocampus; CP = cerebral peduncle; CST = corticospinal tract; dMRI = diffusion-weighted MRI; EC = external capsule; FA = fractional anisotropy; FOR = fornix; FST = fornix / stria terminalis; GCC = genu of corpus callosum; ICP = inferior cerebellar peduncle; ISOVF = isotropic volume fraction; MCP = middle cerebellar peduncle; MD = mean diffusivity; MET = metabolic equivalents; ML = medial lemniscus; NDI = neurite density index; ODI = orientation dispersion index; PCR = posterior corona radiata; PCT = pontine crossing tract; PLIC = posterior limb of internal capsule; PTR = posterior thalamic radiation; RIC = rostral limb of internal capsule; SCC = splenium of corpus callosum; SCP = superior cerebellar peduncle; SCR = superior corona radiata; SFOF = superior fronto-occipital fasciculus; SLF = superior longitudinal fasciculus; SS = sagittal striatum; TAP = tapetum; UF = uncinate fasciculus.

## Supplementary Figure 16: Effects of metabolic equivalents (MET hours) on regional microstructure (Model 3)

**Note:** * p < 0.05; ** p < 0.0007

Corrected p-value derived from correlation matrix of 27 tracts × five diffusion measures in the sensitivity-dataset.

Higher order terms (i.e. quadratic and interaction terms) were only re-entered in consecutive models if they reached statistical significance.

**Abbreviations:** ACR = anterior corona radiata; ALIC = anterior limb of internal capsule; BCC = body of corpus callosum; CCG = cingulum /cingulate gyrus; CH = cingulum hippocampus; CP = cerebral peduncle; CST = corticospinal tract; dMRI = diffusion-weighted MRI; EC = external capsule; FA = fractional anisotropy; FOR = fornix; FST = fornix / stria terminalis; GCC = genu of corpus callosum; ICP = inferior cerebellar peduncle; ISOVF = isotropic volume fraction; MCP = middle cerebellar peduncle; MD = mean diffusivity; MET = metabolic equivalents; ML = medial lemniscus; NDI = neurite density index; ODI = orientation dispersion index; PCR = posterior corona radiata; PCT = pontine crossing tract; PLIC = posterior limb of internal capsule; PTR = posterior thalamic radiation; RIC = rostral limb of internal capsule; SCC = splenium of corpus callosum; SCP = superior cerebellar peduncle; SCR = superior corona radiata; SFOF = superior fronto-occipital fasciculus; SLF = superior longitudinal fasciculus; SS = sagittal striatum; TAP = tapetum; UF = uncinate fasciculus.

## Supplementary Figure 17: Effects of metabolic equivalents (MET hours) on regional microstructure (Model 4)

**Note:** * p < 0.05; ** p < 0.0007

Corrected p-value derived from correlation matrix of 27 tracts × five diffusion measures in the sensitivity-dataset.

Higher order terms (i.e. quadratic and interaction terms) were only re-entered in consecutive models if they reached statistical significance.

**Abbreviations:** ACR = anterior corona radiata; ALIC = anterior limb of internal capsule; BCC = body of corpus callosum; CCG = cingulum /cingulate gyrus; CH = cingulum hippocampus; CP = cerebral peduncle; CST = corticospinal tract; dMRI = diffusion-weighted MRI; EC = external capsule; FA = fractional anisotropy; FOR = fornix; FST = fornix / stria terminalis; GCC = genu of corpus callosum; ICP = inferior cerebellar peduncle; ISOVF = isotropic volume fraction; MCP = middle cerebellar peduncle; MD = mean diffusivity; MET = metabolic equivalents; ML = medial lemniscus; NDI = neurite density index; ODI = orientation dispersion index; PCR = posterior corona radiata; PCT = pontine crossing tract; PLIC = posterior limb of internal capsule; PTR = posterior thalamic radiation; RIC = rostral limb of internal capsule; SCC = splenium of corpus callosum; SCP = superior cerebellar peduncle; SCR = superior corona radiata; SFOF = superior fronto-occipital fasciculus; SLF = superior longitudinal fasciculus; SS = sagittal striatum; TAP = tapetum; UF = uncinate fasciculus.

# Additional analyses

## Supplementary Figure 18: Analyses in total white matter

**Note:** * p < 0.05; ** p < 0.0008

Results shown for the final model (Model 3; controlled for age, sex, education, PA × age, and where applicable for age^2^ and PA^2^).

**Abbreviations:** ACR = anterior corona radiata; ALIC = anterior limb of internal capsule; BCC = body of corpus callosum; CCG = cingulum /cingulate gyrus; CH = cingulum hippocampus; CP = cerebral peduncle; CST = corticospinal tract; EC = external capsule; FA = fractional anisotropy; FOR = fornix; FST = fornix / stria terminalis; GCC = genu of corpus callosum; ICP = inferior cerebellar peduncle; ISOVF = isotropic volume fraction; MCP = middle cerebellar peduncle; MD = mean diffusivity; MET = metabolic equivalents; ML = medial lemniscus; MVPA = moderate-to-vigorous physical activity; NDI = neurite density index; ODI = orientation dispersion index; PCR = posterior corona radiata; PCT = pontine crossing tract; PLIC = posterior limb of internal capsule; PTR = posterior thalamic radiation; RIC = rostral limb of internal capsule; SCC = splenium of corpus callosum; SCP = superior cerebellar peduncle; SCR = superior corona radiata; SFOF = superior fronto-occipital fasciculus; SLF = superior longitudinal fasciculus; SS = sagittal striatum; TAP = tapetum; UF = uncinate fasciculus.

## Supplementary Figure 19: Associations between orientation dispersion index (ODI) and C-reactive protein (CRP)

**Note:** r = Pearson’s correlation coefficient

* p < 0.05; ** p < 0.01; *** p < 0.001.

Overall correlation between global ODI and CRP: r = 0.16 (p < 0.001);

Overall correlation between global extracellular volume fraction and CRP: r = -0.02 (p = 0.18).

**Abbreviations:** ACR = anterior corona radiata; ALIC = anterior limb of internal capsule; BCC = body of corpus callosum; CCG = cingulum /cingulate gyrus; CH = cingulum hippocampus; CP = cerebral peduncle; CST = corticospinal tract; EC = external capsule; FOR = fornix; FST = fornix / stria terminalis; GCC = genu of corpus callosum; ICP = inferior cerebellar peduncle; MCP = middle cerebellar peduncle; ML = medial lemniscus; PCR = posterior corona radiata; PCT = pontine crossing tract; PLIC = posterior limb of internal capsule; PTR = posterior thalamic radiation; RIC = rostral limb of internal capsule; SCC = splenium of corpus callosum; SCP = superior cerebellar peduncle; SCR = superior corona radiata; SFOF = superior fronto-occipital fasciculus; SLF = superior longitudinal fasciculus; SS = sagittal striatum; TAP = tapetum; UF = uncinate fasciculus.

## Supplementary Figure 20: Contributions of variables to cardiometabolic risk components

**A: B:**

**Abbreviations**: DBP = diastolic blood pressure; HDL = high-density lipoprotein; LDL = low-density lipoprotein; SBP = systolic blood pressure; WHR = waist-to-hip ratio.
